# Confidence intervals for rainfall dispersions using the ratio of two coefficients of variation of lognormal distributions with excess zeros

**DOI:** 10.1371/journal.pone.0265875

**Published:** 2022-03-23

**Authors:** Noppadon Yosboonruang, Sa-Aat Niwitpong, Suparat Niwitpong

**Affiliations:** Department of Applied Statistics, Faculty of Applied Science, King Mongkut’s University of Technology North Bangkok, Bangkok, Thailand; Arizona State University, UNITED STATES

## Abstract

Rainfall fluctuation is directly affected by the Earth’s climate change. It can be described using the coefficient of variation (CV). Similarly, the ratio of CVs can be used to compare the rainfall variation between two regions. The ratio of CVs has been widely used in statistical inference in a number of applications. Meanwhile, the confidence interval constructed with this statistic is also of interest. In this paper, confidence intervals for the ratio of two independent CVs of lognormal distributions with excess zeros using the fiducial generalized confidence interval (FGCI), Bayesian methods based on the left-invariant Jeffreys, Jeffreys rule, and uniform priors, and the Wald and Fieller log-likelihood methods are proposed. The results of a simulation study reveal that the highest posterior density (HPD) Bayesian using the Jeffreys rule prior method performed the best in terms of the coverage probability and the average length for almost all cases of small sample size and a large sample size together with a large variance and a small proportion of non-zero values. The performance of the statistic is demonstrated on two rainfall datasets from the central and southern regions in Thailand.

## Introduction

The Earth’s climate is changing due to increased greenhouse gas emission from human activities, and climate change has resulted in dramatic weather events such as heatwaves, heavy rainfall, droughts, etc. Thailand is a country situated in the southeastern region of Asia that is affected by the southwest and northeast monsoons at different times of the year [1]. The country is divided into five regions: North, Northeast, Central, East, and South. Over the past three decades, Thailand has suffered from increased temperatures and fluctuating rainfall. In this study, rainfall is of interest because too much rain causes flooding and too little causes droughts at different times of the year in each area of the country. Rainfall fluctuation can be described using the CV, meaning that the ratio of CVs can be used to compare the rainfall variation between two regions. The lognormal distribution with excess zeros, a mixed distribution of discrete and continuous random variables, has been applied in many studies involving observations with zero values (such as rainfall data) [2–6]. Observations with zero values conform to a binomial distribution whereas the positive values with skewness follow a lognormal distribution. Moreover, the lognormal distribution with excess zeros has been widely used in fields such as fishery surveys [7–9], climatology [10, 11], and medicine [12, 13].

The CV is the ratio of the standard deviation to the mean that is more widely used than the standard deviation when comparing the dispersion in two or more datasets. There have been many applications of the CV to compare the dispersion in datasets. For example, Verrill and Johnson [14] compared the CVs of the xylan percentage for laboratory quality control data in the United States Department of Agriculture forest products laboratory, while Gulhar et al. [15] established confidence intervals for health-related datasets, including birth weight and cigarette smoking. Nam and Kwon [16] applied CVs to estrogen metabolites in blood and urine measurement data. Marek [17] used CVs for mining and ore geology to classify bituminous coal deposits in the Upper Silesian Coal Basin in Poland.

In statistical inference, the confidence intervals for the CV and the function of CVs have attracted the interest of several researchers. Wong and Wu [18] recommended a small sample asymptotic method to construct the confidence intervals for the CV of small sample sizes following normal, gamma, or Weibull distributions. Mahmoudvand and Hassani [19] proposed two confidence intervals for the CV for a normal distribution based on an asymptotically unbiased estimator for the CV that worked well, especially for small sample sizes. Hayter [20] applied confidence intervals (a directional bound and a non-directional upper bound) for the CV of a normal distribution applied to win probabilities. For the ratio of CVs, Nam and Kwon [16] introduced confidence intervals based on the Wald-type, Fieller-type, and log methods, and the method of variance estimate recovery (MOVER) of lognormal distributions. Hasan and Krishnamoorthy [21] suggested MOVER and the fiducial method to establish confidence intervals for small sample sizes from lognormal distributions. Wong and Jiang [22] proposed the Bartlett-corrected likelihood ratio method to construct confidence intervals for lognormal distributions. In addition, Sangnawakij and Niwitpong [23] used the score and Wald methods to construct confidence intervals for the CV and function of CVs with bounded parameter spaces in two gamma distributions for which both proposed methods were evidently suitable. They also constructed confidence intervals using MOVER, the generalized confidence interval (GCI), and the asymptotic confidence interval for CV and the difference between CVs with two parameters exponential distributions of which the GCI was satisfied [24]. Moreover, for a lognormal distribution with excess zeros, Buntao and Niwitpong [25] presented the generalized pivotal approach (GPA) and the closed-form method for obtaining the confidence intervals for the difference between CVs and reported that GPA performed well. Later, the same authors established the confidence intervals for the ratio of CVs under the GPA and MOVER methods, the results revealing that GPA was the most accurate [26]. Recently, Yosboonruang et al. [27] compared GCI and a modified Fletcher method in the confidence interval construction for CV (GCI was the most suitable). After that, they suggested FGCI, comparable with MOVER, to establish the confidence intervals for the CV of a lognormal distribution with excess zeros with three parameters [28]. Moreover, Yosboonruang et al. [29] reported a Bayesian confidence interval compared with FGCI, the results indicating that the Bayesian method performed the best.

In this paper, the ratio of CVs from two lognormal distributions with excess zeros is proposed. Therefore, the concept of FGCI [28], the Bayesian method [29], Wald- and Fieller-type methods [16] were extended to establish confidence intervals for the ratio of two independent CVs from lognormal distributions with excess zeros. The proposed methods for constructing the confidence intervals are presented in the next section. Subsequently, the results of a simulation study are reported to assess the coverage probabilities and average lengths for comparing the proposed methods. Next, application of the proposed methods to rainfall datasets from two regions in Thailand is demonstrated. Last, the paper is brought to a close with a discussion and conclusion.

## Materials and methods

Let $X_{ij}=(X_{i1},X_{i2},\ldots ,X_{in_{i}})$, for *i* = 1, 2, *j* = 1, 2, …, *n*_*i*_, be a semi-continuous random sample that conforms a lognormal distribution with excess zeros with the probability of zero values *δ*_*i*,0_, mean *μ*_*i*_, and variance $\sigma_{i}^{2}$, denoted by $X_{ij}∼\Delta (\delta_{i,0},\mu_{i},\sigma_{i}^{2})$. The zero observations have a binomial distribution, while the non-zero observations follow a lognormal distribution. The numbers of zero and non-zero observations are defined as *n*_*i*,0_ and *n*_*i*,1_, respectively, where *n*_*i*_ = *n*_*i*,0_ + *n*_*i*,1_. This leads to the distribution function of *X*_*ij*_:

$$\begin{array}{c} F(x_{ij;\delta_{i,0},\mu_{i},\sigma_{i}^{2}})=\{\begin{array}{cc} \delta_{i,0} & ;x_{ij}=0\\ \delta_{i,0}+(1-\delta_{i,0})\;H\;(x_{ij};\mu_{i},\sigma_{i}^{2}) & ;x_{ij}>0, \end{array} \end{array}$$ (1)

where *δ*_*i*,0_ = *P*(*x*_*ij*_ = 0), *n*_*i*,0_ ∼ *B*(*n*_*i*_, *δ*_*i*,0_) [6], and $H(x_{ij};\mu_{i},\sigma_{i}^{2})$ is a lognormal cumulative distribution function [11], so ln *X*_*ij*_ follows a normal distribution with mean *μ*_*i*_ and variance $\sigma_{i}^{2}$ for *X*_*ij*_ > 0. Thus, the probability density function of *X*_*ij*_ can be expressed as

$$\begin{array}{c} \begin{array}{cc} & f(x_{ij};\delta_{i,0},\mu_{i},\sigma_{i}^{2})\\ & =\delta_{i,0}{\text{I}}_{0}[x_{ij}]+(1-\delta_{i,0})\frac{1}{x_{ij}\sqrt{2\pi }\sigma_{i}}\;\text{exp}\;[-\frac{1}{2}{\left(\frac{\text{ln}\;x_{ij}-\mu_{i}}{\sigma_{i}}\right)}^{2}]{\text{I}}_{\left(0,\infty \right)}[x_{ij}] \end{array} \end{array}$$ (2)

such that if *x*_*ij*_ = 0, then I_0_ [*x*_*ij*_] = 1 and I_(0,∞)_ [*x*_*ij*_] = 0, and if *x*_*ij*_ > 0, then I_(0,∞)_ [*x*_*ij*_] = 1. According to Aitchison [30], the population mean and variance of *X*_*ij*_ are $\mu_{X_{ij}}=\delta_{i,1}\;\text{exp}\;(\mu_{i}+\sigma_{i}^{2}/2)$ and $\sigma_{X_{ij}}^{2}=\delta_{i,1}\;\text{exp}\;(2\mu_{i}+\sigma_{i}^{2})[\;\text{exp}\;(\sigma_{i}^{2})-\delta_{i,1}]$, respectively, where *δ*_*i*,1_ = 1 − *δ*_*i*,0_. Thus, the CV of *X*_*ij*_ can be defined as

$$\begin{array}{c} \text{CV}(X_{ij})=\eta_{i}=\sqrt{\frac{\;\text{exp}\;(\sigma_{i}^{2})-\delta_{i,1}}{\delta_{i,1}}}. \end{array}$$ (3)

The aim here is to construct the confidence interval for the ratio of the CVs:

$$\begin{array}{c} \phi =\frac{\sqrt{\frac{\;\text{exp}(\sigma_{1}^{2})-\delta_{1,1}}{\delta_{1,1}}}}{\sqrt{\frac{\;\text{exp}(\sigma_{2}^{2})-\delta_{2,1}}{\delta_{2,1}}}}. \end{array}$$ (4)

In accordance with a lognormal distribution with excess zeros, the maximum likelihood estimators (MLEs) of parameters *δ*_*i*,1_, *δ*_*i*,0_, and *μ*_*i*_ are ${\hat{\delta }}_{i,1}=n_{i,1}/n_{i}$, ${\hat{\delta }}_{i,0}=n_{i,0}/n_{i}$, and ${\hat{\mu }}_{i}=\sum_{i=1}^{n_{i,1}}\text{ln}\;x_{ij}/n_{i,1}$, respectively. For $\sigma_{i}^{2}$, the unbiased estimator is ${\hat{\sigma }}_{i}^{2}=\sum_{i=1}^{n_{i,1}}{\left(\text{ln}\;x_{ij}-{\hat{\mu }}_{i}\right)}^{2}/(n_{i,1}-1)$. The approaches used to construct the confidence intervals are in the following subsections.

### The fiducial generalized confidence interval

Fiducial inference was initially suggested by Fisher [31], whereupon Hannig [32] and Li, Zhou, and Tian [33] introduced the generalized fiducial quantities (GFQs) for *δ*_*i*,1_ and $\sigma_{i}^{2}$, which are respectively

$$\begin{array}{c} R_{\delta_{i,1}}∼\frac{1}{2}Beta\;(n_{i,1},n_{i,0}+1)+\frac{1}{2}Beta\;(n_{i,1}+1,n_{i,0}) \end{array}$$ (5)

and

$$\begin{array}{c} R_{\sigma_{i}^{2}}=\frac{(n_{i,1}-1){\hat{\sigma }}_{i}^{2}}{U_{i}}, \end{array}$$ (6)

where $U_{i}∼\chi_{n_{i,1}-1}^{2}$.

Moreover, there have been several studies using FGCI to construct confidence intervals [28, 29, 32, 34–37]. From Eqs (5) and (6), the GFQ for *η*_*i*_ is defined as

$$\begin{array}{c} R_{\eta_{i}}=\sqrt{\frac{\;\text{exp}\;(R_{\sigma_{i}^{2}})-R_{\delta_{i,1}}}{R_{\delta_{i,1}}}}. \end{array}$$ (7)

The approach in this study points toward constructing the confidence interval for the ratio of CVs. Thus, the GFQ for *ϕ* is in the form

$$\begin{array}{c} R_{\phi }=\frac{\sqrt{\frac{\;\text{exp}(R_{\sigma_{1}^{2}})-R_{\delta_{1,1}}}{R_{\delta_{1,1}}}}}{\sqrt{\frac{\;\text{exp}(R_{\sigma_{2}^{2}})-R_{\delta_{2,1}}}{R_{\delta_{2,1}}}}}. \end{array}$$ (8)

Therefore, the 100(1 − *α*)% confidence interval for *ϕ* is

$$\begin{array}{c} {\text{CI}}_{\phi }^{\text{FGCI}}=[R_{\phi ,l},R_{\phi ,u}]=[R_{\phi }(\alpha /2),R_{\phi }(1-\alpha /2)], \end{array}$$ (9)

where *R*_*ϕ*_(*α*/2) and *R*_*ϕ*_(1 − *α*/2) are the (*α*/2)-th and (1 − *α*/2)-th percentiles of *R*_*ϕ*_, respectively.

**Algorithm 1**

  **for** *k* = 1 : *M* **do**

   Generate datasets **x**_*ij*_, for *i* = 1, 2, *j* = 1, 2, …, *n*_*i*_, from the lognormal distributions with excess zeros;

   Calculate ${\hat{\delta }}_{i,1}$ and ${\hat{\sigma }}_{i}^{2}$;

   **for** *l* = 1 : *m* **do**

    Generate *Beta* (*n*_*i*,1_, *n*_*i*,0_ + 1) and *Beta* (*n*_*i*,1_ + 1, *n*_*i*,0_);

    Calculate $R_{\delta_{i,1}}$, $R_{\sigma_{i}^{2}}$, and *R*_*ϕ*_;

   **end for**

   Calculate the (*α*/2)-th and (1 − *α*/2)-th percentiles of *R*_*ϕ*_

  **end for**

### Bayesian methods

The probability density function of a lognormal distribution with excess zeros (Eq (2)) has unknown parameters *δ*_*i*,0_, *μ*_*i*_ and $\sigma_{i}^{2}$. The joint likelihood function is defined as

$$\begin{array}{c} L(\delta_{i,0},\mu_{i},\sigma_{i}^{2}|x_{ij})\propto \prod_{i=1}^{2}\{\delta_{i,0}^{n_{i,0}}{\left(1-\delta_{i,0}\right)}^{n_{i,1}}\prod_{j=1}^{n_{i,1}}\frac{1}{\sigma_{i}}\;\text{exp}\;[-\frac{1}{2\sigma_{i}^{2}}{\left(\text{ln}\;x_{ij}-\mu_{i}\right)}^{2}]\}. \end{array}$$ (10)

Since we are interested in the ratio of the CVs, then the Fisher information matrix of the unknown parameters $\left(\delta_{1,0},\mu_{1},\sigma_{1}^{2},\delta_{2,0},\mu_{2},\sigma_{2}^{2}\right)$ computed by the second-order derivative of the log-likelihood function can be expressed as

$$\begin{array}{c} \begin{array}{cc} & I(\delta_{1,0},\mu_{1},\sigma_{1}^{2},\delta_{2,0},\mu_{2},\sigma_{2}^{2})\\ & =\text{diag}\;[\begin{array}{cccccc} \frac{n_{1}}{\delta_{1,0}(1-\delta_{1,0})} & \frac{n_{1,1}}{\sigma_{1}^{2}} & \frac{n_{1}(1-\delta_{1,0})}{2{\left(\sigma_{1}^{2}\right)}^{2}} & \frac{n_{2}}{\delta_{2,0}(1-\delta_{2,0})} & \frac{n_{2,1}}{\sigma_{2}^{2}} & \frac{n_{2}(1-\delta_{2,0})}{2{\left(\sigma_{2}^{2}\right)}^{2}} \end{array}]. \end{array} \end{array}$$ (11)

Subsequently, the equitailed confidence intervals and the HPD intervals are constructed for the left-invariant Jeffreys, the Jeffreys rule, and the uniform priors.

#### The left-invariant Jeffreys prior

Because the lognormal distribution with excess zeros is a combination of binomial and lognormal distributions, the Jeffreys priors for *δ*_*i*,0_ and $\sigma_{i}^{2}$ are computed under these distributions. According to Jeffreys [38] and Ghosh et al. [39], the invariant Jeffreys prior is obtained using a Fisher information matrix (*I*(*θ*)), which is given as $p(\theta )=\sqrt{\left|I(\theta )\right|}$.

Because the left-invariant Jeffreys prior is non-informative, the Jeffreys invariant prior for a binomial proportion (*δ*_*i*,0_) is given by

$$\begin{array}{c} p(\delta_{i,0})=\sqrt{\left|I(\delta_{})\right|}\propto \delta_{i,0}^{-\frac{1}{2}}{\left(1-\delta_{i,0}\right)}^{-\frac{1}{2}}. \end{array}$$ (12)

This leads to the posterior distribution of *δ*_*i*,0_ as

$$\begin{array}{c} p(\delta_{i,0}|n_{i,0})\propto \delta_{i,0}^{n_{i,0}-\frac{1}{2}}{\left(1-\delta_{i,0}\right)}^{n_{i,1}-\frac{1}{2}}, \end{array}$$ (13)

which is a beta distribution with parameters *n*_*i*,0_ + 1/2 and *n*_*i*,1_ + 1/2, denoted by *δ*_*i*,0_ ∣ *n*_*i*,0_ ∼ *Beta* (*n*_*i*,0_ + 1/2, *n*_*i*,1_ + 1/2). Similarly for the lognormal distribution, the left-invariant Jeffreys prior for $\sigma_{i}^{2}$, which is $p(\sigma_{i}^{2})=1/\sigma_{i}^{2}$ [39]. Subsequently, by combining the prior distributions of *δ*_*i*,0_ and $\sigma_{i}^{2}$, we obtain $p(\delta_{i,0},\sigma_{i}^{2})\propto \sigma_{i}^{-2}\delta_{i,0}^{-\frac{1}{2}}{\left(1-\delta_{i,0}\right)}^{-\frac{1}{2}}$ for a lognormal distribution with excess zeros. Accordingly, the joint posterior density function for a lognormal distribution with excess zeros is written as

$$\begin{array}{c} \begin{array}{cc} p(\delta_{i,0},\sigma_{i}^{2}|x_{ij})= & \prod_{i=1}^{2}\{\frac{1}{Beta\;(n_{i,0}+\frac{1}{2},n_{i,1}+\frac{1}{2})}\delta_{i,0}^{n_{i,0}-\frac{1}{2}}{\left(1-\delta_{i,0}\right)}^{n_{i,1}-\frac{1}{2}}\frac{1}{\sqrt{2\pi }\frac{\sigma_{i}}{\sqrt{n_{i,1}}}}\\ & \times \;\text{exp}\;[-\frac{1}{2\frac{\sigma_{i}^{2}}{n_{i,1}}}{\left(\mu_{i}-{\hat{\mu }}_{i}\right)}^{2}]\frac{{\left[\frac{(n_{i,1}-1){\hat{\sigma }}_{i}^{2}}{2}\right]}^{\frac{n_{i,1}-1}{2}}}{\Gamma (\frac{n_{i,1}-1}{2})}{\left(\sigma_{i}^{2}\right)}^{-1-\frac{n_{i,1}-1}{2}}\\ & \times \;\text{exp}\;[-\frac{(n_{i,1}-1){\hat{\sigma }}_{i}^{2}}{2\sigma_{i}^{2}}]\}, \end{array} \end{array}$$ (14)

where ${\hat{\mu }}_{i}=\sum_{j=1}^{n_{i,1}}\text{ln}\;x_{ij}/n_{i,1}$ and ${\hat{\sigma }}_{i}^{2}=\sum_{j=1}^{n_{i,1}}{\left(\text{ln}\;x_{ij}-{\hat{\mu }}_{i}\right)}^{2}/(n_{i,1}-1)$. Therefore, the posterior distribution of *δ*_*i*,0_ is a beta distribution with parameters *n*_*i*,0_ + 1/2 and *n*_*i*,1_ + 1/2, denoted by *δ*_*i*,0_∣*x*_*ij*_ ∼ *Beta* (*n*_*i*,0_ + 1/2, *n*_*i*,1_ + 1/2). Similarly, the posterior distribution of $\sigma_{i}^{2}$ is an inverse gamma distribution with parameters (*n*_*i*,1_ − 1)/2 and $(n_{i,1}-1){\hat{\sigma }}_{i}^{2}/2$, denoted by $\sigma_{i}^{2}|x_{ij}∼IG[(n_{i,1}-1)/2,(n_{i,1}-1){\hat{\sigma }}_{i}^{2}/2]$.

#### The Jeffreys rule prior

The Jeffreys rule prior, which was previously referred to as the square root of the determinant of the Fisher information for a binomial proportion, is $p(\delta_{i,0})\propto \delta_{i,0}^{-\frac{1}{2}}{\left(1-\delta_{i,0}\right)}^{\frac{1}{2}}$ and for $\sigma_{i}^{2}$ from a lognormal distribution is $p(\sigma_{i}^{2})\propto \sigma_{i}^{-3}$ [40]. According to the CV of the lognormal distribution with excess zeros as in Eq (3), the parameters are independent, then the Jeffreys rule prior for $\left(\delta_{i,0},\sigma_{i}^{2}\right)$ is $p(\delta_{i,0},\sigma_{i}^{2})\propto \sigma_{i}^{-3}\delta_{i,0}^{-\frac{1}{2}}{\left(1-\delta_{i,0}\right)}^{\frac{1}{2}}$. Therefore, the joint posterior density function is defined as

$$\begin{array}{c} \begin{array}{cc} & p(\delta_{i,0},\sigma_{i}^{2}|x_{ij})=\\ & \prod_{i=1}^{2}\{\frac{1}{Beta\;(n_{i,0}+\frac{1}{2},n_{i,1}+\frac{3}{2})}\delta_{i,0}^{n_{i,0}-\frac{1}{2}}{\left(1-\delta_{i,0}\right)}^{n_{i,1}+\frac{1}{2}}\frac{1}{\sqrt{2\pi }\frac{\sigma_{i}}{\sqrt{n_{i,1}}}}\\ & \times \;\text{exp}\;[-\frac{1}{2\frac{\sigma_{i}^{2}}{n_{i,1}}}{\left(\mu_{i}-{\hat{\mu }}_{i}\right)}^{2}]\frac{{\left[\frac{n_{i,1}{\hat{\sigma }}_{i}^{2}}{2}\right]}^{\frac{n_{i,1}}{2}}}{\Gamma (\frac{n_{i,1}}{2})}{\left(\sigma_{i}^{2}\right)}^{-1-\frac{n_{i,1}}{2}}\;\text{exp}\;[-\frac{n_{i,1}{\hat{\sigma }}_{i}^{2}}{2\sigma_{i}^{2}}]\}, \end{array} \end{array}$$ (15)

where ${\hat{\mu }}_{i}=\sum_{j=1}^{n_{i,1}}\text{ln}\;x_{ij}/n_{i,1}$ and ${\hat{\sigma }}_{i}^{2}=\sum_{j=1}^{n_{i,1}}{\left(\text{ln}\;x_{ij}-{\hat{\mu }}_{i}\right)}^{2}/(n_{i,1}-1)$. Subsequently, the posterior densities of *δ*_*i*,0_ and $\sigma_{i}^{2}$ follow a beta distribution, *Beta* (*n*_*i*,0_ + 1/2, *n*_*i*,1_ + 3/2), and an inverse gamma distribution, $IG(n_{i,1}/2,n_{i,1}{\hat{\sigma }}_{i}^{2}/2)$, respectively.

#### The uniform prior

For the uniform prior, the prior probability is a constant function whereby all possible values are equally likely to be a *priori* [41, 42]. Accordingly, for binomial and lognormal distributions, the uniform priors of *δ*_*i*,0_ and $\sigma_{i}^{2}$ are proportional to 1 [43, 44], which implies that the uniform prior for a lognormal distribution with excess zeros is $p(\delta_{i,0},\sigma_{i}^{2})\propto 1$. The joint posterior distribution for a lognormal distribution with excess zeros is given by

$$\begin{array}{c} \begin{array}{cc} p(\delta_{i,0},\sigma_{i}^{2}|x_{ij})= & \prod_{i=1}^{2}\{\frac{1}{Beta\;(n_{i,0}+1,n_{i,1}+1)}\delta_{i,0}^{n_{i,0}}{\left(1-\delta_{i,0}\right)}^{n_{i,1}}\frac{1}{\sqrt{2\pi }\frac{\sigma_{i}}{\sqrt{n_{i,1}}}}\\ & \times \;\text{exp}\;[-\frac{1}{2\frac{\sigma_{i}^{2}}{n_{i,1}}}{\left(\mu_{i}-{\hat{\mu }}_{i}\right)}^{2}]\frac{{\left[\frac{(n_{i,1}-2){\hat{\sigma }}_{i}^{2}}{2}\right]}^{\frac{n_{i,1}-2}{2}}}{\Gamma (\frac{n_{i,1}-2}{2})}{\left(\sigma_{i}^{2}\right)}^{-1-\frac{n_{i,1}-2}{2}}\\ & \times \;\text{exp}\;[-\frac{(n_{i,1}-2){\hat{\sigma }}_{i}^{2}}{2\sigma_{i}^{2}}]\}, \end{array} \end{array}$$ (16)

where ${\hat{\mu }}_{i}=\sum_{j=1}^{n_{i,1}}\text{ln}\;x_{ij}/n_{i,1}$ and ${\hat{\sigma }}_{i}^{2}=\sum_{j=1}^{n_{i,1}}{\left(\text{ln}\;x_{ij}-{\hat{\mu }}_{i}\right)}^{2}/(n_{i,1}-1)$. Thus, the posterior distribution for *δ*_*i*,0_ follows a beta distribution, *Beta* (*n*_*i*,0_ + 1, *n*_*i*,1_ + 1), and that of $\sigma_{i}^{2}$ is an inverse gamma distribution, $\sigma_{i}^{2}|x_{ij}∼IG[(n_{i,1}-2)/2,(n_{i,1}-2){\hat{\sigma }}_{i}^{2}/2]$.

The posterior distributions of *δ*_*i*,0_ and $\sigma_{i}^{2}$ can be replaced by following Eq (4), and then the equitailed confidence intervals and HPD intervals are constructed by imposing Algorithm 2.

**Algorithm 2**

  **for** *k* = 1 : *M* **do**

   Generate datasets **x**_*ij*_, for *i* = 1, 2, *j* = 1, 2, …, *n*_*i*_, from the lognormal distributions with excess zeros;

   Calculate ${\hat{\delta }}_{i,1}$ and ${\hat{\sigma }}_{i}^{2}$;

   **for** *l* = 1 : *m* **do**

    Generate the posterior densities of *δ*_*i*,0_ ∣ *x*_*ij*_,

   • left-invariant Jeffreys prior: *δ*_*i*,0_∣*x*_*ij*_ ∼ *Beta* (*n*_*i*,0_ + 1/2, *n*_*i*,1_ + 1/2),

   • Jeffreys rule prior: *δ*_*i*,0_∣*x*_*ij*_ ∼ *Beta* (*n*_*i*,0_ + 1/2, *n*_*i*,1_ + 3/2),

   • uniform prior: *δ*_*i*,0_∣*x*_*ij*_ ∼ *Beta* (*n*_*i*,0_ + 1, *n*_*i*,1_ + 1);

    Generate the posterior densities of $\sigma_{i}^{2}|x_{ij}$,

   • left-invariant Jeffreys prior: $\sigma_{i}^{2}|x_{ij}∼IG[(n_{i,1}-1)/2,(n_{i,1}-1){\hat{\sigma }}_{i}^{2}/2]$,

   • Jeffreys rule prior: $\sigma_{i}^{2}|x_{ij}∼IG(n_{i,1}/2,n_{i,1}{\hat{\sigma }}_{i}^{2}/2)$,

   • uniform prior: $\sigma_{i}^{2}|x_{ij}∼IG[(n_{i,1}-2)/2,(n_{i,1}-2){\hat{\sigma }}_{i}^{2}/2]$;

    Calculate *ϕ* from Eq (4) for the left-invariant Jeffreys, the Jeffreys rule, and the uniform priors;

   **end for**

   Construct HPD intervals and equitailed confidence intervals for *ϕ* based on the left-invariant Jeffreys, the Jeffreys rule, and the uniform priors

  **end for**

### The Wald log-likelihood method

According to Eq (10), the log-likelihood function is

$$\begin{array}{c} \begin{array}{cc} \text{ln}\;L\propto & n_{1,0}\;\text{ln}\;\delta_{1,0}+n_{2,0}\;\text{ln}\;\delta_{2,0}+n_{1,1}\;\text{ln}\;(1-\delta_{1,0})+n_{2,1}\;\text{ln}\;(1-\delta_{2,0})\\ & -\frac{1}{2}\{[n_{1,1}\;\text{ln}\;\sigma_{1}^{2}+\frac{1}{\sigma_{1}^{2}}\sum_{j=1}^{n_{1,1}}{\left(\text{ln}\;x_{1j}-\mu_{1}\right)}^{2}]\\ & +[n_{2,1}\;\text{ln}\;\sigma_{2}^{2}+\frac{1}{\sigma_{2}^{2}}\sum_{j=1}^{n_{2,1}}{\left(\text{ln}\;x_{2j}-\mu_{2}\right)}^{2}]\}. \end{array} \end{array}$$ (17)

From Eq (4), the parameter of interest is *ϕ*. Subsequently, the log-likelihood function is reparameterized in terms of *ϕ* by substituting *η*_1_ = *ϕη*_2_, $\sigma_{1}^{2}=\text{ln}\;(1-\delta_{1,0})+\text{ln}\;(\phi^{2}\eta_{2}^{2}+1)$, and $\sigma_{2}^{2}=\text{ln}\;(1-\delta_{2,0})+\text{ln}\;(\eta_{2}^{2}+1)$ into Eq (17) as follows

$$\begin{array}{c} \begin{array}{cc} \text{ln}\;L\propto & n_{1,0}\;\text{ln}\;\delta_{1,0}+n_{2,0}\;\text{ln}\;\delta_{2,0}+n_{1,1}\;\text{ln}\;(1-\delta_{1,0})+n_{2,1}\;\text{ln}\;(1-\delta_{2,0})\\ & -\frac{1}{2}\{[n_{1,1}\;\text{ln}\;(\text{ln}\;(1-\delta_{1,0})+\text{ln}\;(\phi^{2}\eta_{2}^{2}+1))+\frac{\sum_{j=1}^{n_{1,1}}{\left(\text{ln}\;x_{1j}-\mu_{1}\right)}^{2}}{\text{ln}\;(1-\delta_{1,0})+\text{ln}\;(\phi^{2}\eta_{2}^{2}+1)}]\\ & +[n_{2,1}\;\text{ln}\;(\text{ln}\;(1-\delta_{2,0})+\text{ln}\;(\eta_{2}^{2}+1))+\frac{\sum_{j=1}^{n_{2,1}}{\left(\text{ln}\;x_{2j}-\mu_{2}\right)}^{2}}{\text{ln}\;(1-\delta_{2,0})+\text{ln}\;(\eta_{2}^{2}+1)}]\}. \end{array} \end{array}$$ (18)

**Theorem 1**. *Let* $X_{ij}∼\Delta (\delta_{i,0},\mu_{i},\sigma_{i}^{2})$, *where i* = 1, 2, *j* = 1, 2, …, *n*_*i*_. *Let* $\text{ln}\;X_{ij}∼N(\mu_{i},\sigma_{i}^{2})$, *for X*_*ij*_ > 0. *Likewise, let* $\phi ={\left\{[(\;\text{exp}\;(\sigma_{1}^{2})-\delta_{1,0})/\delta_{1,0}]/[(\;\text{exp}\;(\sigma_{2}^{2})-\delta_{2,0})/\delta_{2,0}]\right\}}^{1/2}$, *where δ*_*i*,1_ = 1 − *δ*_*i*,0_ *for i* = 1, 2, *be the ratio of CVs of lognormal distributions with excess zeros. The unrestricted MLEs of δ*_*i*,1_, *μ*_*i*_, *and* $\sigma_{i}^{2}$ *are* ${\hat{\delta }}_{i,1}=n_{i,1}/n_{i}$, ${\hat{\mu }}_{i}=\sum_{j=1}^{n_{i,1}}\text{ln}\;x_{ij}/n_{i,1}$, *and* ${\hat{\sigma }}_{i}^{2}=\sum_{j=1}^{n_{i,1}}{\left(\text{ln}\;x_{ij}-{\hat{\mu }}_{i}\right)}^{2}/n_{i}$, *respectively. Consequently*, ${\hat{\eta }}_{i}={\left[(\;\text{exp}\;({\hat{\sigma }}_{i}^{2})-{\hat{\delta }}_{i,1})/{\hat{\delta }}_{i,1}\right]}^{1/2}$ *and* $\hat{\phi }={\left\{[(\;\text{exp}\;({\hat{\sigma }}_{1}^{2})-{\hat{\delta }}_{1,1})/{\hat{\delta }}_{1,1}]/[(\;\text{exp}\;({\hat{\sigma }}_{2}^{2})-{\hat{\delta }}_{2,1})/{\hat{\delta }}_{2,1}]\right\}}^{1/2}$. *Therefore, the asymptotic variance of* $\hat{\phi }$ *is*

$$\begin{array}{c} Var(\hat{\phi })=\frac{n_{1,1}\phi^{4}{\left[\sigma_{2}^{2}(\eta_{2}^{2}+1)\right]}^{2}+n_{2,1}{\left[\sigma_{1}^{2}(\eta_{1}^{2}+1)\right]}^{2}}{2n_{1,1}n_{2,1}\eta_{1}^{2}\eta_{2}^{2}}. \end{array}$$ (19)

*Proof*. Following Nam and Kwon [16], since the MLE of $\sigma_{i}^{2}$ is ${\hat{\sigma }}_{i}^{2}=\sum_{j=1}^{n_{i,1}}{\left(\text{ln}\;x_{ij}-{\hat{\mu }}_{i}\right)}^{2}/n_{i}$, where ${\hat{\mu }}_{i}=\sum_{j=1}^{n_{i,1}}\text{ln}\;x_{ij}/n_{i,1}$ for *i* = 1, 2, then the log-likelihood function for reparameterization from Eq (17) can be written as

$$\begin{array}{c} \begin{array}{cc} \text{ln}\;L\propto & n_{1,0}\;\text{ln}\;\delta_{1,0}+n_{2,0}\;\text{ln}\;\delta_{2,0}+n_{1,1}\;\text{ln}\;(1-\delta_{1,0})+n_{2,1}\;\text{ln}\;(1-\delta_{2,0})\\ & -\frac{1}{2}\{[n_{1,1}\;\text{ln}\;(\text{ln}\;(1-\delta_{1,0})+\text{ln}\;(\phi^{2}\eta_{2}^{2}+1))+\frac{n_{1,1}{\hat{\sigma }}_{1}^{2}}{\text{ln}\;(1-\delta_{1,0})+\text{ln}\;(\phi^{2}\eta_{2}^{2}+1)}]\\ & +[n_{2,1}\;\text{ln}\;(\text{ln}\;(1-\delta_{2,0})+\text{ln}\;(\eta_{2}^{2}+1))+\frac{n_{2,1}{\hat{\sigma }}_{2}^{2}}{\text{ln}\;(1-\delta_{2,0})+\text{ln}\;(\eta_{2}^{2}+1)}]\}. \end{array} \end{array}$$

The asymptotic variance of $\hat{\phi }$ is obtained using the Fisher information which is also written as

$$\begin{array}{c} I(\theta )=-E(\frac{\partial^{2}\;\text{ln}\;L}{\partial \theta^{2}}). \end{array}$$

By the second-order partial derivative, the Fisher information elements are

$$\begin{array}{c} \begin{array}{cc} & I_{11}=-E(\frac{\partial^{2}\;\text{ln}\;L}{\partial \phi^{2}})=\frac{2n_{1,1}\eta_{1}^{2}\eta_{2}^{2}}{{\left[\sigma_{1}^{2}(\eta_{1}^{2}+1)\right]}^{2}}\\ & I_{22}=-E(\frac{\partial^{2}\;\text{ln}\;L}{\partial \eta_{2}^{2}})=\frac{2n_{1,1}\phi^{2}\eta_{1}^{2}}{{\left[\sigma_{1}^{2}(\eta_{1}^{2}+1)\right]}^{2}}+\frac{2n_{2,1}\eta_{2}^{2}}{{\left[\sigma_{2}^{2}(\eta_{2}^{2}+1)\right]}^{2}}\\ & I_{33}=-E(\frac{\partial^{2}\;\text{ln}\;L}{\partial \delta_{1,0}^{2}})=\frac{n_{1}}{\delta_{1,0}(1-\delta_{1,0})}\\ & I_{44}=-E(\frac{\partial^{2}\;\text{ln}\;L}{\partial \delta_{2,0}^{2}})=\frac{n_{2}}{\delta_{2,0}(1-\delta_{2,0})}\\ & I_{55}=-E(\frac{\partial^{2}\;\text{ln}\;L}{\partial \mu_{1}^{2}})=\frac{n_{1,1}}{\sigma_{1}^{2}}\\ & I_{66}=-E(\frac{\partial^{2}\;\text{ln}\;L}{\partial \mu_{2}^{2}})=\frac{n_{2,1}}{\sigma_{2}^{2}}\\ & I_{12}=I_{21}=-E(\frac{\partial^{2}\;\text{ln}\;L}{\partial \phi \partial \eta_{2}})=\frac{2n_{1,1}\eta_{1}^{3}}{{\left[\sigma_{1}^{2}(\eta_{1}^{2}+1)\right]}^{2}} \end{array} \end{array}$$

and the other elements are zeros. By the left-hand block of the matrix $I_{n}^{-1}(\theta )$, *I*^11^, the asymptotic variance of $\hat{\phi }$ is

$$\begin{array}{c} \begin{array}{cc} Var(\hat{\phi }) & =I^{11}={\left(I_{11}-\frac{I_{12}^{2}}{I_{21}}\right)}^{-1}\\ & =\frac{n_{1,1}\phi^{4}{\left[\sigma_{2}^{2}(\eta_{2}^{2}+1)\right]}^{2}+n_{2,1}{\left[\sigma_{1}^{2}(\eta_{1}^{2}+1)\right]}^{2}}{2n_{1,1}n_{2,1}\eta_{1}^{2}\eta_{2}^{2}}, \end{array} \end{array}$$

where *η*_1_ = *ϕη*_2_ and $\sigma_{i}^{2}=\text{ln}\;\delta_{i,1}+\text{ln}\;(\eta_{i}^{2}+1)$ for *i* = 1, 2.

Following the MLEs of the parameters $\delta_{i,1}={\hat{\delta }}_{i,1}$, $\eta_{i}={\hat{\eta }}_{i}$, $\sigma_{i}^{2}={\hat{\sigma }}_{i}^{2}$, for *i* = 1, 2, and $\phi =\hat{\phi }$, the variance estimate for $\hat{\phi }$ is

$$\begin{array}{c} \hat{Var}(\hat{\phi })=\frac{n_{1,1}{\hat{\phi }}^{4}{\left[{\hat{\sigma }}_{2}^{2}({\hat{\eta }}_{2}^{2}+1)\right]}^{2}+n_{2,1}{\left[{\hat{\sigma }}_{1}^{2}({\hat{\eta }}_{1}^{2}+1)\right]}^{2}}{2n_{1,1}n_{2,1}{\hat{\eta }}_{1}^{2}{\hat{\eta }}_{2}^{2}}. \end{array}$$ (20)

The asymptotically standard normal distribution is

$$\begin{array}{c} Z_{1}=\frac{\hat{\phi }-\phi }{\sqrt{\hat{Var}(\hat{\phi })}}∼N(0,1). \end{array}$$ (21)

Therefore, the 100(1 − *α*)% two-sided confidence interval for *ϕ* based on the Wald log-likelihood method is

$$\begin{array}{c} \phi =\hat{\phi }\pm z_{1-\alpha /2}\sqrt{\hat{Var}(\hat{\phi })}, \end{array}$$ (22)

where *z*_1−*α*/2_ is the (1 − *α*/2)-th percentile of the standard normal distribution.

### The Fieller log-likelihood method

Following Eq (17) and since $\sigma_{i}^{2}=\text{ln}\;(1-\delta_{i,0})+\text{ln}\;(\eta_{i}^{2}+1)$, the log-likelihood function can be written as

$$\begin{array}{c} \begin{array}{cc} \text{ln}\;L\propto & n_{1,0}\;\text{ln}\;\delta_{1,0}+n_{2,0}\;\text{ln}\;\delta_{2,0}+n_{1,1}\;\text{ln}\;(1-\delta_{1,0})+n_{2,1}\;\text{ln}\;(1-\delta_{2,0})\\ & -\frac{1}{2}\{[n_{1,1}\;\text{ln}\;(\text{ln}\;(1-\delta_{1,0})+\text{ln}\;(\eta_{1}^{2}+1))+\frac{\sum_{j=1}^{n_{1,1}}{\left(\text{ln}\;x_{1j}-\mu_{1}\right)}^{2}}{\text{ln}\;(1-\delta_{1,0})+\text{ln}\;(\eta_{1}^{2}+1)}]\\ & +[n_{2,1}\;\text{ln}\;(\text{ln}\;(1-\delta_{2,0})+\text{ln}\;(\eta_{2}^{2}+1))+\frac{\sum_{j=1}^{n_{2,1}}{\left(\text{ln}\;x_{2j}-\mu_{2}\right)}^{2}}{\text{ln}\;(1-\delta_{2,0})+\text{ln}\;(\eta_{2}^{2}+1)}]\}. \end{array} \end{array}$$ (23)

**Theorem 2**. *Let* $X_{ij}∼\Delta (\delta_{i,0},\mu_{i},\sigma_{i}^{2})$, *where i* = 1, 2, *j* = 1, 2, …, *n*_*i*_, *and let* $\text{ln}\;X_{ij}∼N(\mu_{i},\sigma_{i}^{2})$, *for X*_*ij*_ > 0. *The CV of a lognormal distribution with excess zeros is* $\eta_{i}={\left\{[\;\text{exp}\;(\sigma_{i}^{2})-\delta_{i,1}]/\delta_{i,1}\right\}}^{1/2}$, *where δ*_*i*,1_ = 1 − *δ*_*i*,0_ *for i* = 1, 2. *Let* ${\hat{\delta }}_{i,1}=n_{i,1}/n_{i}$, ${\hat{\mu }}_{i}=\sum_{j=1}^{n_{i,1}}\text{ln}\;x_{ij}/n_{i,1}$, *and* ${\hat{\sigma }}_{i}^{2}=\sum_{j=1}^{n_{i,1}}{\left(\text{ln}\;x_{ij}-{\hat{\mu }}_{i}\right)}^{2}/n_{i}$ *be the unrestricted MLEs of δ*_*i*,1_, *μ*_*i*_, *and* $\sigma_{i}^{2}$, *respectively. Likewise*, ${\hat{\eta }}_{i}={\left\{[\;\text{exp}\;({\hat{\sigma }}_{i}^{2})-{\hat{\delta }}_{i,1}]/{\hat{\delta }}_{i,1}\right\}}^{1/2}$. *Therefore, the asymptotic variance of* ${\hat{\eta }}_{i}$ *are*

$$\begin{array}{c} Var({\hat{\eta }}_{i})=\frac{{\left\{[\text{ln}\;(1-\delta_{i,0})+\text{ln}\;(\eta_{i}^{2}+1)](\eta_{i}^{2}+1)\right\}}^{2}}{2n_{i,1}\eta_{i}^{2}}, \end{array}$$ (24)

*for i* = 1, 2.

*Proof*. The MLEs of the parameters are obtained from the first-order derivative of Eq (23) as ${\hat{\delta }}_{i,1}=n_{i,1}/n_{i}$, ${\hat{\mu }}_{i}=\sum_{j=1}^{n_{i,1}}\text{ln}\;x_{ij}/n_{i,1}$, and ${\hat{\sigma }}_{i}^{2}=\sum_{j=1}^{n_{i,1}}{\left(\text{ln}\;x_{ij}-{\hat{\mu }}_{i}\right)}^{2}/n_{i}$. Thus, ${\hat{\eta }}_{i}={\left\{[\;\text{exp}\;({\hat{\sigma }}_{i}^{2})-{\hat{\delta }}_{i,1}]/{\hat{\delta }}_{i,1}\right\}}^{1/2}$, for *i* = 1, 2. Similarly to Theorem 1, the elements of the Fisher information matrix are

$$\begin{array}{c} \begin{array}{cc} I_{mn} & =-E(\frac{\partial^{2}\;\text{ln}\;L}{\partial \eta_{m}\partial \eta_{n}})\\ & =\frac{2n_{i,1}\eta_{i}^{2}}{{\left\{[\text{ln}\;(1-\delta_{i,0})+\text{ln}\;(\eta_{i}^{2}+1)](\eta_{i}^{2}+1)\right\}}^{2}} \end{array} \end{array}$$

for *m* = *n* = 1, 2, *i* = 1, 2, *I*_*mn*_ for *m* = *n* = 3, 4, 5, 6 follows from Theorem 1 when $\sigma_{i}^{2}=\text{ln}\;(1-\delta_{i,0})+\text{ln}\;(\eta_{i}^{2}+1)$ and *I*_*mn*_ = 0 for *m*, *n* = 1, 2, …, 6 and *m* ≠ *n*. The asymptotic variances of ${\hat{\eta }}_{1}$ and ${\hat{\eta }}_{2}$ are

$$\begin{array}{c} Var({\hat{\eta }}_{1})=I^{11}=I_{11}^{-1}=\frac{{\left\{[\text{ln}\;(1-\delta_{1,0})+\text{ln}\;(\eta_{1}^{2}+1)](\eta_{1}^{2}+1)\right\}}^{2}}{2n_{1,1}\eta_{1}^{2}} \end{array}$$

and

$$\begin{array}{c} Var({\hat{\eta }}_{2})=I^{22}=I_{22}^{-1}=\frac{{\left\{[\text{ln}\;(1-\delta_{2,0})+\text{ln}\;(\eta_{2}^{2}+1)](\eta_{2}^{2}+1)\right\}}^{2}}{2n_{2,1}\eta_{2}^{2}}. \end{array}$$

Since *δ*_*i*,1_ = 1 − *δ*_*i*,0_ and the MLEs of *δ*_*i*,1_ and $\sigma_{i}^{2}$ are ${\hat{\delta }}_{i,1}$ and ${\hat{\sigma }}_{i}^{2}$, respectively, then the estimated variance of ${\hat{\eta }}_{i}$ is

$$\begin{array}{c} \hat{Var}({\hat{\eta }}_{i})=\frac{{\left[{\hat{\sigma }}_{i}^{2}({\hat{\eta }}_{i}^{2}+1)\right]}^{2}}{2n_{i,1}{\hat{\eta }}_{i}^{2}}, \end{array}$$ (25)

where ${\hat{\sigma }}_{i}^{2}=\text{ln}\;{\hat{\delta }}_{i,1}+\text{ln}\;({\hat{\eta }}_{i}^{2}+1)$, for *i* = 1, 2. According to Fieller [45], the statistic

$$\begin{array}{c} Z_{2}=\frac{{\hat{\eta }}_{1}-\phi {\hat{\eta }}_{2}}{\sqrt{\hat{Var}({\hat{\eta }}_{1})+\phi^{2}\hat{Var}({\hat{\eta }}_{2})}}∼N(0,1). \end{array}$$ (26)

Therefore, the 100(1 − *α*)% two-sided confidence interval for *ϕ* based on the Fieller log-likelihood method is

$$\begin{array}{c} \phi =\frac{{\hat{\eta }}_{1}{\hat{\eta }}_{2}\pm \sqrt{{\left({\hat{\eta }}_{1}{\hat{\eta }}_{2}\right)}^{2}-[{\hat{\eta }}_{1}^{2}-z_{1-\alpha /2}^{2}\hat{Var}({\hat{\eta }}_{1})][{\hat{\eta }}_{2}^{2}-z_{1-\alpha /2}^{2}\hat{Var}({\hat{\eta }}_{2})]}}{{\hat{\eta }}_{2}^{2}-z_{1-\alpha /2}^{2}\hat{Var}({\hat{\eta }}_{2})}. \end{array}$$ (27)

where *z*_1−*α*/2_ is the (1 − *α*/2)-th percentile of a standard normal distribution.

Note that: The variance of the estimator for ratio of CVs from Theorem 1 and the variance of the estimator for CV from Theorem 2 are equal to the variances which are reported by Nam and Kwon [16] when *δ*_1,1_, *δ*_2,1_ = 1.

**Algorithm 3**

  **for** *k* = 1 : *M* **do**

   Generate datasets **x**_*ij*_, for *i* = 1, 2, *j* = 1, 2, …, *n*_*i*_, from the lognormal distributions with excess zeros;

   Calculate ${\hat{\delta }}_{i,1}$ and ${\hat{\sigma }}_{i}^{2}$;

   Calculate $\hat{Var}(\hat{\phi })$ from Eq (20) and $\hat{Var}({\hat{\eta }}_{i})$ from Eq (25);

   Construct two-sided confidence intervals for *ϕ* based on the Wald log-likelihood and Fieller log-likelihood methods

  **end for**

## Results and discussion

### Simulation studies

Simulation studies were conducted to compare the performances of the methods used to construct the confidence intervals using FGCI, the Bayesian methods (the left-invariant Jeffreys, the Jeffreys rule, and the uniform priors), and the Wald and Fieller log-likelihood methods. The optimal method was the one with a coverage probability equal to or greater than the nominal confidence level of 0.95 together with the shortest average length. Following Wu and Hsieh [46], cases that were expected to have non-zero values of less than 10 were not considered. Sample sizes (*n*_*i*_), *δ*_*i*,1_, and $\sigma_{i}^{2}$ were set as reported in Table 1. For all of the simulations, 15,000 runs were generated and 5,000 replicates were defined for the FGCI and Bayesian methods via Monte Carlo simulation using RStudio version 1.1.463.

**Table 1:** The coverage probabilities and average lengths of 95% two-sided confidence intervals for the ratio of CVs of lognormal distributions with excess zeros.

*n*_1_: *n*_2_	*δ*_1_: *δ*_2_	$\sigma_{1}^{2}:\sigma_{2}^{2}$	Coverage Probabilities (Average Lengths)								
			FGCI	Equitailed			HPD			Wald	Fieller
				B-LIJ	B-JR	B-U	B-LIJ	B-JR	B-U		
25:25	0.5:0.5	0.5:0.5	**0.9654**	**0.9835**	**0.9799**	**0.9859**	**0.9872**	**0.9844**	**0.9893**	0.9061	**0.9516**
			(1.3234)	(1.4643)	(1.3937)	(1.5297)	(1.3608)	(1.3037)	(1.4109)	(0.7803)	**(0.8304)**
		0.5:1.0	**0.9586**	**0.9719**	**0.9671**	**0.9760**	**0.9725**	**0.9679**	**0.9760**	0.9330	0.9475
			(1.1076)	(1.1805)	(1.1220)	(1.2369)	(1.1129)	**(1.0636)**	(1.1579)	(0.7528)	(0.9368)
		1.0:2.0	**0.9535**	**0.9573**	0.9489	**0.9640**	**0.9555**	0.9496	**0.9628**	0.9407	0.8281
			(1.7466)	(1.7645)	(1.6379)	(1.9158)	**(1.4734)**	(1.3975)	(1.5569)	(1.0605)	(1.1056)
		2.0:2.0	**0.9514**	**0.9559**	0.9477	**0.9636**	**0.9573**	**0.9514**	**0.9627**	0.9127	0.8597
			(10.9684)	(11.3842)	(9.5735)	(14.3922)	(6.2440)	**(5.5766)**	(7.1173)	(2.5565)	(9.6299)
	0.8:0.8	0.5:0.5	**0.9561**	**0.9675**	**0.9631**	**0.9717**	**0.9711**	**0.9667**	**0.9754**	0.9215	**0.9579**
			(1.1465)	(1.2155)	(1.1818)	(1.2381)	(1.1582)	(1.1287)	(1.1775)	(0.8839)	(0.9510)
		0.5:1.0	**0.9555**	**0.9618**	**0.9566**	**0.9665**	**0.9623**	**0.9580**	**0.9679**	0.9319	**0.9654**
			(0.8710)	(0.9016)	(0.8747)	(0.9254)	(0.8672)	(0.8431)	(0.8887)	(0.7100)	(0.8447)
		1.0:2.0	**0.9538**	**0.9529**	0.9477	**0.9584**	0.9495	0.9455	**0.9545**	0.9360	0.9438
			(1.1656)	(1.1758)	(1.1340)	(1.2198)	(1.0753)	(1.0432)	(1.1082)	(0.8974)	(1.8105)
		2.0:2.0	**0.9501**	**0.9503**	0.9445	**0.9554**	**0.9511**	0.9463	**0.9552**	0.9147	**0.9723**
			(4.1302)	(4.2305)	(3.9862)	(4.5007)	(3.2591)	(3.1220)	(3.4102)	(2.1424)	(3.9999)
25:50	0.5:0.5	0.5:0.5	**0.9631**	**0.9804**	**0.9769**	**0.9821**	**0.9776**	**0.9725**	**0.9805**	0.8478	0.8945
			(1.1354)	(1.2463)	(1.1768)	(1.3098)	(1.1396)	**(1.0852)**	(1.1826)	(0.6463)	(0.6637)
		0.5:1.0	**0.9561**	**0.9735**	**0.9692**	**0.9765**	**0.9714**	**0.9653**	**0.9746**	0.8943	**0.9739**
			(0.9068)	(0.9743)	(0.9210)	(1.0214)	(0.9043)	(0.8615)	(0.9387)	(0.5858)	**(0.6421)**
		1.0:2.0	**0.9513**	**0.9600**	**0.9535**	**0.9651**	**0.9583**	**0.9517**	**0.9632**	0.9133	**0.9937**
			(1.4721)	(1.5075)	(1.3869)	(1.6444)	(1.2475)	(1.1733)	(1.3248)	(0.8232)	**(1.1562)**
		2.0:2.0	**0.9548**	**0.9577**	0.9499	**0.9635**	**0.9543**	0.9473	**0.9605**	0.8630	**0.9895**
			(10.3410)	(10.0671)	(8.3466)	(12.8534)	(5.5641)	(4.9297)	(6.4182)	(2.1428)	**(2.7247)**
	0.8:0.8	0.5:0.5	**0.9553**	**0.9683**	**0.9653**	**0.9718**	**0.9706**	**0.9674**	**0.9773**	0.9011	0.9323
			(0.9915)	(1.0529)	(1.0218)	(1.0811)	(0.9942)	**(0.9679)**	(1.0177)	(0.7378)	(0.7615)
		0.5:1.0	**0.9549**	**0.9660**	**0.9621**	**0.9697**	**0.9650**	**0.9602**	**0.9719**	0.9099	**0.9621**
			(0.7237)	(0.7571)	(0.7351)	(0.7791)	(0.7208)	(0.7016)	(0.7395)	(0.5652)	**(0.6086)**
		1.0:2.0	**0.9503**	**0.9548**	**0.9513**	**0.9586**	**0.9581**	**0.9543**	**0.9619**	0.9227	**0.9943**
			(0.9815)	(0.9901)	(0.9555)	(1.0304)	(0.8970)	(0.8709)	(0.9267)	(0.7072)	**(0.8692)**
		2.0:2.0	**0.9500**	**0.9530**	0.9481	**0.9577**	**0.9581**	**0.9534**	**0.9623**	0.8895	**0.9829**
			(3.7856)	(3.7697)	(3.5480)	(4.0469)	(2.8749)	(2.7497)	(3.0279)	(1.7976)	**(2.1417)**
25:100	0.5:0.5	0.5:0.5	**0.9592**	**0.9803**	**0.9756**	**0.9830**	**0.9736**	**0.9659**	**0.9778**	0.7939	0.8203
			(1.0311)	(1.1495)	(1.0758)	(1.2122)	(1.0394)	(0.9820)	(1.0809)	(0.5785)	(0.5853)
		0.5:1.0	**0.9543**	**0.9745**	**0.9689**	**0.9788**	**0.9685**	**0.9582**	**0.9719**	0.8447	0.9081
			(0.7945)	(0.8644)	(0.8109)	(0.9090)	(0.7896)	(0.7474)	(0.8201)	(0.4895)	(0.5095)
		1.0:2.0	**0.9526**	**0.9637**	**0.9580**	**0.9684**	**0.9611**	**0.9515**	**0.9649**	0.8653	**0.9762**
			(1.3343)	(1.3555)	(1.2358)	(1.4897)	(1.0972)	(1.0246)	(1.1716)	(0.6752)	(0.7689)
		2.0:2.0	**0.9516**	**0.9553**	0.9462	**0.9624**	0.9495	0.9401	**0.9568**	0.8298	0.9202
			(9.5088)	(10.2704)	(8.2102)	(13.2450)	(5.3998)	(4.7107)	(6.2762)	(1.9127)	(2.1279)
	0.8:0.8	0.5:0.5	**0.9548**	**0.9670**	**0.9643**	**0.9713**	**0.9675**	**0.9619**	**0.9740**	0.8671	0.8899
			(0.9126)	(0.9673)	(0.9359)	(0.9988)	(0.9077)	(0.8810)	(0.9333)	(0.6615)	(0.6709)
		0.5:1.0	**0.9554**	**0.9627**	**0.9597**	**0.9654**	**0.9625**	**0.9575**	**0.9697**	0.8796	0.9219
			(0.6407)	(0.6715)	(0.6502)	(0.6935)	(0.6332)	(0.6151)	(0.6517)	(0.4826)	(0.4985)
		1.0:2.0	**0.9507**	**0.9555**	**0.9520**	**0.9595**	**0.9584**	**0.9555**	**0.9647**	0.8897	**0.9595**
			(0.8574)	(0.8692)	(0.8360)	(0.9081)	(0.7766)	(0.7520)	(0.8048)	(0.5893)	(0.6416)
		2.0:2.0	**0.9525**	**0.9512**	0.9471	**0.9551**	**0.9550**	**0.9503**	**0.9611**	0.8657	0.9255
			(3.5906)	(3.5590)	(3.3357)	(3.8322)	(2.6723)	(2.5466)	(2.8233)	(1.6200)	(1.7423)
50:50	0.2:0.2	0.5:0.5	**0.9675**	**0.9885**	**0.9869**	**0.9902**	**0.9904**	**0.9889**	**0.9913**	0.8934	0.9381
			(1.4029)	(1.5611)	(1.4427)	(1.6879)	(1.4160)	**(1.3269)**	(1.5007)	(0.6473)	(0.6791)
		0.5:1.0	**0.9625**	**0.9778**	**0.9743**	**0.9803**	**0.9755**	**0.9729**	**0.9767**	0.9166	0.9160
			(1.2525)	(1.3672)	(1.2661)	(1.4770)	(1.2576)	**(1.1810)**	(1.3302)	(0.7180)	(1.0312)
		1.0:2.0	**0.9524**	**0.9617**	**0.9527**	**0.9697**	**0.9631**	**0.9555**	**0.9689**	0.9392	0.7470
			(2.4409)	(2.4928)	(2.1804)	(2.9603)	(1.8501)	**(1.7018)**	(2.0355)	(1.1185)	(1.7014)
		2.0:2.0	**0.9533**	**0.9613**	**0.9512**	**0.9697**	**0.9571**	**0.9500**	**0.9647**	0.9063	0.8193
			(40.7663)	(36.0997)	(21.8269)	(65.5736)	(11.6103)	**(9.0939)**	(15.9990)	(2.8913)	(5.2774)
	0.5:0.5	0.5:0.5	**0.9598**	**0.9823**	**0.9807**	**0.9835**	**0.9835**	**0.9827**	**0.9849**	0.9105	0.9327
			**(0.7841)**	(0.8854)	(0.8689)	(0.8957)	(0.8595)	(0.8443)	(0.8689)	(0.5544)	(0.5713)
		0.5:1.0	**0.9549**	**0.9679**	**0.9648**	**0.9701**	**0.9689**	**0.9671**	**0.9715**	0.9281	0.9475
			**(0.6816)**	(0.7365)	(0.7209)	(0.7477)	(0.7210)	(0.7062)	(0.7316)	(0.5435)	(0.6019)
		1.0:2.0	**0.9508**	**0.9583**	**0.9545**	**0.9616**	**0.9589**	**0.9546**	**0.9619**	0.9427	**0.9540**
			(0.9225)	(0.9453)	(0.9228)	(0.9676)	(0.8966)	**(0.8774)**	(0.9154)	(0.7578)	(1.0524)
		2.0:2.0	**0.9500**	**0.9528**	0.9483	**0.9561**	**0.9556**	**0.9521**	**0.9587**	0.9277	**0.9967**
			(2.7610)	(2.8132)	(2.7145)	(2.9196)	(2.3746)	**(2.3092)**	(2.4431)	(1.7547)	(2.8522)
	0.8:0.8	0.5:0.5	**0.9563**	**0.9671**	**0.9643**	**0.9697**	**0.9677**	**0.9658**	**0.9701**	0.9279	0.9477
			**(0.7258)**	(0.7740)	(0.7646)	(0.7788)	(0.7565)	(0.7477)	(0.7610)	(0.6165)	(0.6389)
		0.5:1.0	**0.9532**	**0.9570**	**0.9547**	**0.9606**	**0.9585**	**0.9565**	**0.9615**	0.9365	**0.9583**
			(0.5606)	(0.5818)	(0.5743)	(0.5881)	(0.5715)	(0.5644)	(0.5777)	(0.4986)	**(0.5417)**
		1.0:2.0	**0.9529**	**0.9500**	0.9467	**0.9524**	**0.9503**	0.9482	**0.9537**	0.9443	**0.9637**
			(0.7013)	(0.7099)	(0.7000)	(0.7197)	**(0.6869)**	(0.6779)	(0.6960)	(0.6267)	(0.7872)
		2.0:2.0	**0.9501**	**0.9502**	0.9473	**0.9525**	**0.9549**	**0.9532**	**0.9572**	0.9347	**0.9973**
			(1.8696)	(1.8701)	(1.8372)	(1.9029)	(1.7035)	**(1.6779)**	(1.7292)	(1.4471)	(1.7632)
50:100	0.2:0.2	0.5:0.5	**0.9658**	**0.9882**	**0.9855**	**0.9891**	**0.9873**	**0.9830**	**0.9879**	0.8433	0.8855
			(1.2156)	(1.3690)	(1.2439)	(1.4802)	(1.2081)	**(1.1179)**	(1.2694)	(0.5533)	(0.5645)
		0.5:1.0	**0.9630**	**0.9821**	**0.9784**	**0.9839**	**0.9817**	**0.9752**	**0.9824**	0.9113	**0.9712**
			(1.0392)	(1.1399)	(1.0410)	(1.2270)	(1.0282)	(0.9547)	(1.0788)	(0.5707)	**(0.6263)**
		1.0:2.0	**0.9549**	**0.9655**	**0.9599**	**0.9703**	**0.9662**	**0.9575**	**0.9695**	0.9301	**0.9753**
			(2.0945)	(2.2044)	(1.8812)	(2.6662)	(1.6118)	(1.4499)	(1.7906)	(0.8727)	**(0.5866)**
		2.0:2.0	**0.9538**	**0.9571**	0.9485	**0.9642**	**0.9548**	0.9444	**0.9610**	0.8528	**0.9797**
			(67.8516)	(61.5960)	(34.1736)	(164.6780)	(13.8966)	(10.3774)	(22.4465)	(2.3299)	**(3.0886)**
	0.5:0.5	0.5:0.5	**0.9635**	**0.9821**	**0.9806**	**0.9829**	**0.9805**	**0.9774**	**0.9819**	0.8793	0.9045
			**(0.6718)**	(0.7570)	(0.7399)	(0.7661)	(0.7309)	(0.7154)	(0.7383)	(0.4717)	(0.4779)
		0.5:1.0	**0.9559**	**0.9739**	**0.9723**	**0.9752**	**0.9715**	**0.9679**	**0.9737**	0.9097	**0.9511**
			(0.5556)	(0.6052)	(0.5918)	(0.6130)	(0.5882)	(0.5758)	(0.5951)	(0.4297)	**(0.4496)**
		1.0:2.0	**0.9505**	**0.9586**	**0.9567**	**0.9611**	**0.9617**	**0.9573**	**0.9643**	0.9262	**0.9882**
			(0.7576)	(0.7811)	(0.7609)	(0.7979)	(0.7341)	(0.7172)	(0.7473)	(0.5904)	**(0.6832)**
		2.0:2.0	**0.9503**	**0.9529**	**0.9502**	**0.9563**	**0.9583**	**0.9540**	**0.9609**	0.9050	**0.9792**
			(2.5209)	(2.5261)	(2.4280)	(2.6227)	(2.0993)	(2.0352)	(2.1614)	(1.4996)	**(1.6864)**
	0.8:0.8	0.5:0.5	**0.9535**	**0.9659**	**0.9639**	**0.9668**	**0.9657**	**0.9630**	**0.9684**	0.9121	0.9310
			**(0.6263)**	(0.6669)	(0.6586)	(0.6734)	(0.6496)	(0.6418)	(0.6555)	(0.5218)	(0.5300)
		0.5:1.0	**0.9529**	**0.9593**	**0.9575**	**0.9616**	**0.9575**	**0.9550**	**0.9609**	0.9172	0.9489
			**(0.4624)**	(0.4817)	(0.4756)	(0.4872)	(0.4710)	(0.4654)	(0.4763)	(0.3999)	(0.4148)
		1.0:2.0	**0.9531**	**0.9518**	0.9495	**0.9543**	**0.9517**	0.9489	**0.9546**	0.9286	**0.9765**
			(0.5747)	(0.5844)	(0.5767)	(0.5925)	(0.5615)	(0.5547)	(0.5688)	(0.4967)	**(0.5467)**
		2.0:2.0	**0.9529**	**0.9518**	0.9485	**0.9531**	**0.9599**	**0.9575**	**0.9618**	0.9165	**0.9705**
			(1.6544)	(1.6684)	(1.6388)	(1.7006)	(1.4996)	(1.4773)	(1.5243)	(1.2334)	**(1.3380)**
100:100	0.2:0.2	0.5:0.5	**0.9637**	**0.9885**	**0.9875**	**0.9888**	**0.9908**	**0.9904**	**0.9917**	0.8920	0.9182
			(0.7637)	(0.8899)	(0.8673)	(0.9033)	(0.8618)	(0.8411)	(0.8735)	(0.4694)	(0.4801)
		0.5:1.0	**0.9616**	**0.9755**	**0.9732**	**0.9781**	**0.9785**	**0.9772**	**0.9804**	0.9227	0.9277
			(0.7215)	(0.8006)	(0.7793)	(0.8150)	(0.7834)	(0.7633)	(0.7971)	(0.5276)	(0.5839)
		1.0:2.0	**0.9513**	**0.9607**	**0.9556**	**0.9641**	**0.9615**	**0.9579**	**0.9639**	0.9433	0.9315
			(1.0450)	(1.0894)	(1.0542)	(1.1220)	(1.0187)	(0.9904)	(1.0444)	(0.8018)	(1.3385)
		2.0:2.0	**0.9522**	**0.9589**	**0.9543**	**0.9627**	**0.9599**	**0.9571**	**0.9631**	0.9270	**0.9835**
			(3.4830)	(3.5748)	(3.3877)	(3.7837)	(2.8333)	(2.7227)	(2.9520)	(1.8358)	(2.4826)
	0.5:0.5	0.5:0.5	**0.9562**	**0.9829**	**0.9820**	**0.9836**	**0.9831**	**0.9820**	**0.9835**	0.9056	0.9177
			**(0.5149)**	(0.5854)	(0.5805)	(0.5878)	(0.5765)	(0.5719)	(0.5788)	(0.3961)	(0.4021)
		0.5:1.0	**0.9543**	**0.9725**	**0.9710**	**0.9739**	**0.9706**	**0.9701**	**0.9723**	0.9307	0.9420
			**(0.4555)**	(0.4926)	(0.4879)	(0.4958)	(0.4873)	(0.4826)	(0.4904)	(0.3895)	(0.4093)
		1.0:2.0	**0.9513**	**0.9558**	**0.9546**	**0.9588**	**0.9581**	**0.9555**	**0.9603**	0.9457	**0.9545**
			(0.5917)	(0.6083)	(0.6020)	(0.6142)	(0.5957)	**(0.5897)**	(0.6013)	(0.5379)	(0.6305)
		2.0:2.0	**0.9523**	**0.9529**	**0.9510**	**0.9550**	**0.9590**	**0.9585**	**0.9622**	0.9387	**0.9918**
			(1.4645)	(1.4979)	(1.4795)	(1.5164)	(1.4009)	(1.3850)	(1.4158)	(1.1982)	**(1.3712)**
	0.8:0.8	0.5:0.5	**0.9520**	**0.9681**	**0.9674**	**0.9699**	**0.9682**	**0.9667**	**0.9688**	0.9345	0.9434
			**(0.4892)**	(0.5242)	(0.5212)	(0.5255)	(0.5174)	(0.5146)	(0.5188)	(0.4333)	(0.4410)
		0.5:1.0	**0.9507**	**0.9599**	**0.9582**	**0.9603**	**0.9601**	**0.9579**	**0.9611**	0.9379	0.9499
			**(0.3813)**	(0.3973)	(0.3948)	(0.3992)	(0.3932)	(0.3908)	(0.3951)	(0.3531)	(0.3677)
		1.0:2.0	**0.9512**	0.9498	0.9492	**0.9518**	**0.9501**	0.9487	**0.9522**	0.9437	**0.9623**
			(0.4669)	(0.4715)	(0.4685)	(0.4746)	**(0.4642)**	(0.4612)	(0.4671)	(0.4406)	(0.4900)
		2.0:2.0	**0.9510**	**0.9505**	0.9485	**0.9524**	**0.9554**	**0.9527**	**0.9563**	0.9415	**0.9786**
			(1.1197)	(1.1283)	(1.1201)	(1.1361)	(1.0821)	**(1.0748)**	(1.0892)	(0.9968)	(1.0932)

**Fig 1:**
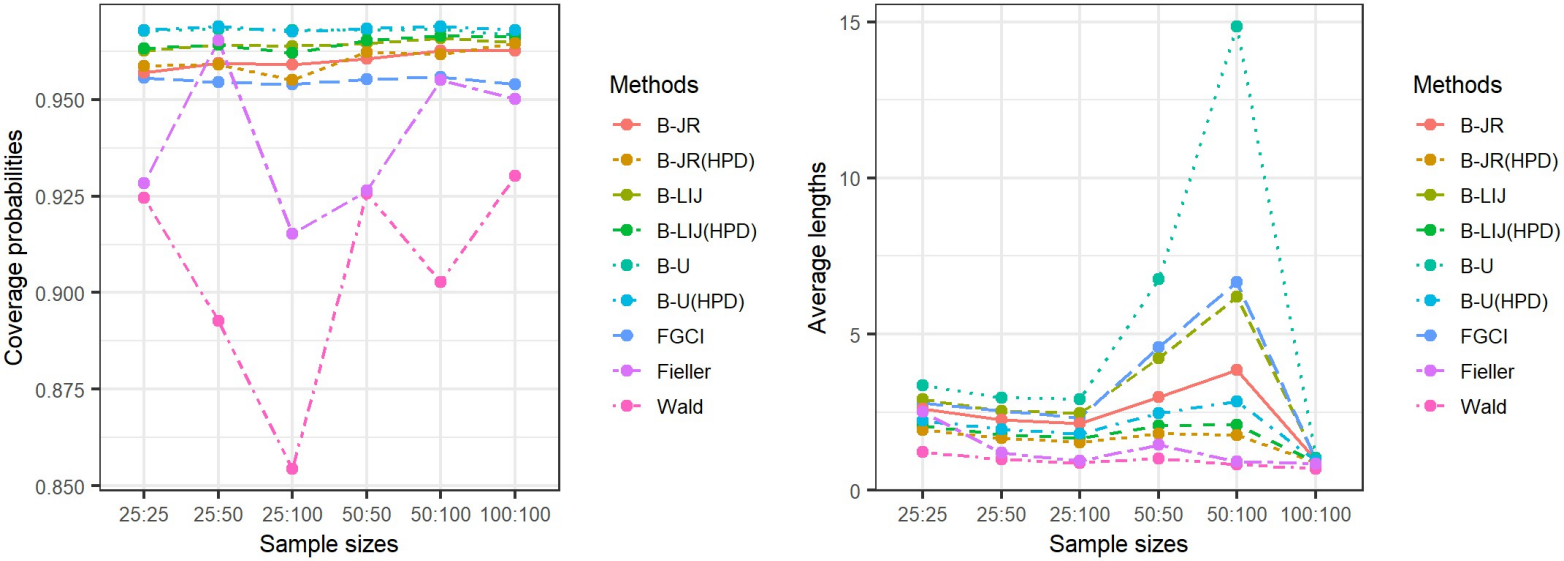
Line graphs for comparing the coverage probabilities (CP) and average lengths (AL) of all methods in cases of the different sample sizes.

**Fig 2:**
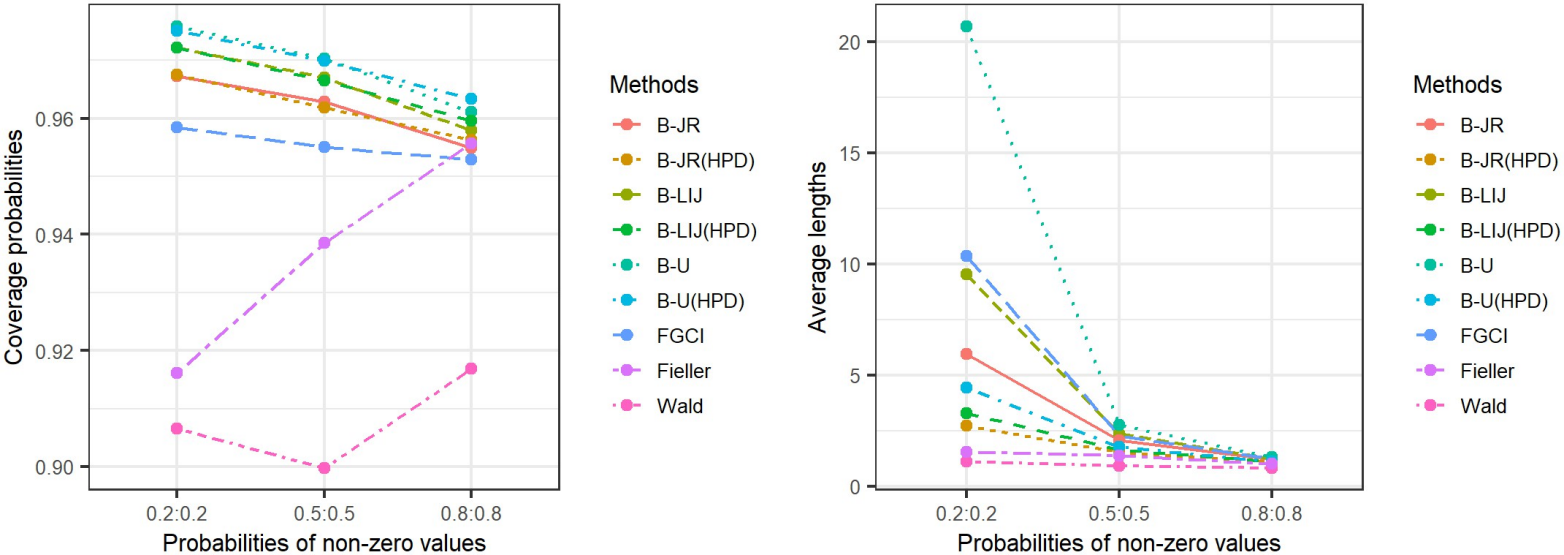
Line graphs for comparing the coverage probabilities (CP) and average lengths (AL) of all methods in cases of the different probabilities of non-zero values.

**Fig 3:**
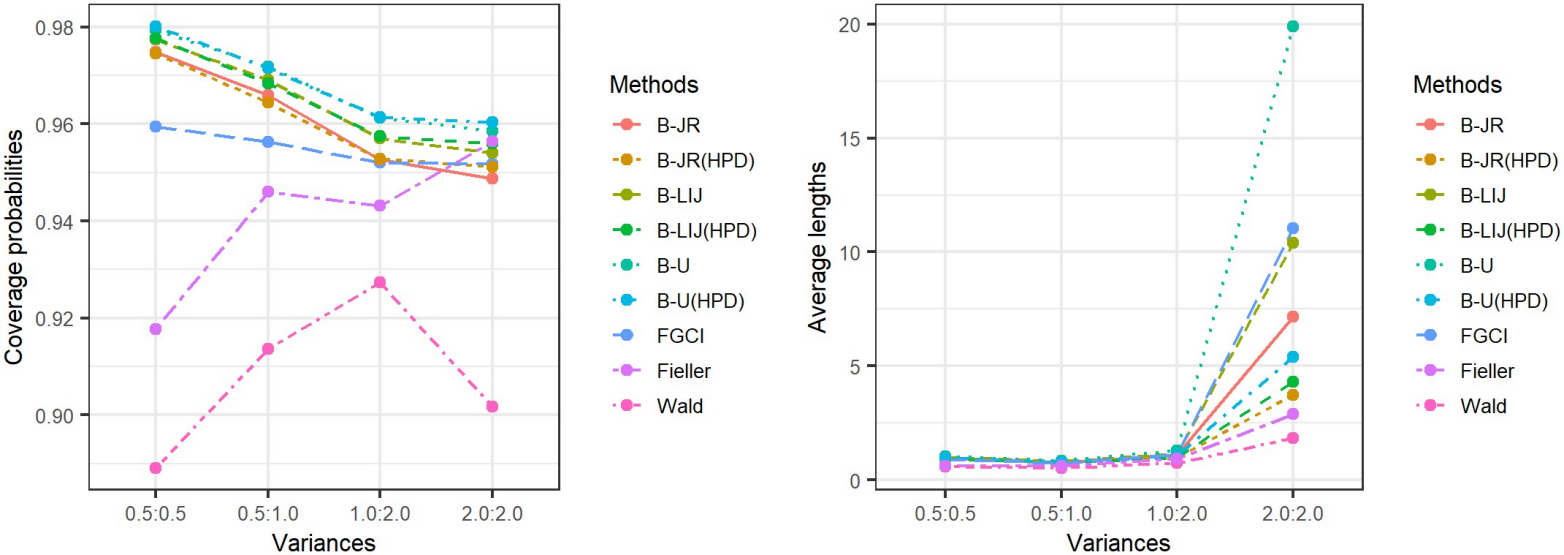
Line graphs for comparing the coverage probabilities (CP) and average lengths (AL) of all methods in cases of the different variances.

Table 1 and Figs 1–3 present the coverage probabilities and average lengths of the confidence intervals for the various methods. The results show that the coverage probabilities of FGCI were consistently close to the nominal confidence level of 0.95 for all cases. The coverage probabilities of the Bayesian method using the uniform prior (B-U) for both the equitailed confidence interval and HPD interval were greater than or close to the nominal confidence level of 0.95 for all cases. The Bayesian methods using the left-invariant Jeffreys (B-LIJ) and the Jeffreys rule (B-JR) priors based on equitailed confidence intervals and HPD intervals attained coverage probabilities greater than or close to the nominal confidence level of 0.95 in almost every case. However, those attained by the Wald log-likelihood method were less than the nominal confidence level 0.95 for all cases whereas those produced by the Fieller log-likelihood method were greater than or close to the nominal confidence level 0.95 in some cases.

The average lengths of B-JR based on the HPD interval was the shortest for most of the cases when the sample size was small (*n*_1_ and/or *n*_2_ = 25). For large sample sizes (*n*_1_, *n*_2_ = 50, 100), B-JR based on the HPD interval had mainly narrow average lengths for the cases of *δ*_1,1_, *δ*_2,1_ = 0.2 for all variances and *δ*_1,1_, *δ*_2,1_ = 0.5, 0.8 together with $\sigma_{1}^{2}$, $\sigma_{2}^{2}=1,2$, while those of FGCI were the shortest for cases with small variance(s) ($\sigma_{1}^{2}$ and/or $\sigma_{2}^{2}=0.5$).

### An empirical example

**Fig 4:**
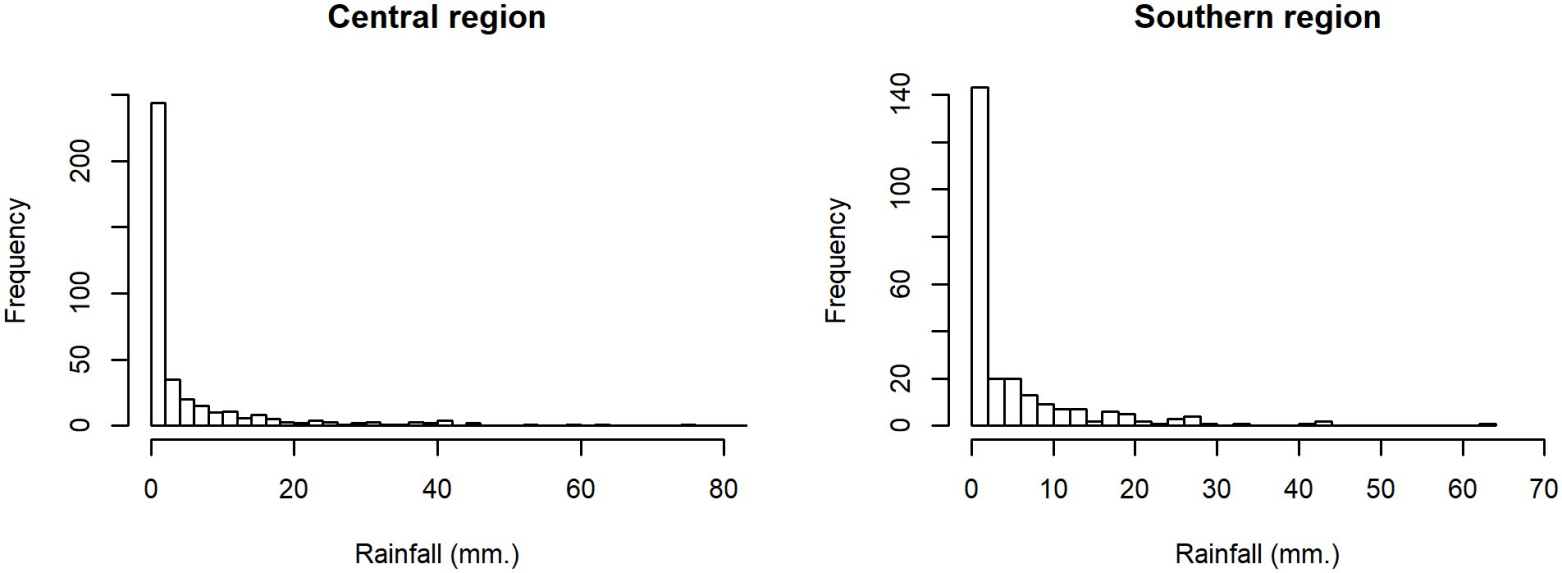
Density of rainfall data sets from central and southern regions in Thailand.

**Fig 5:**
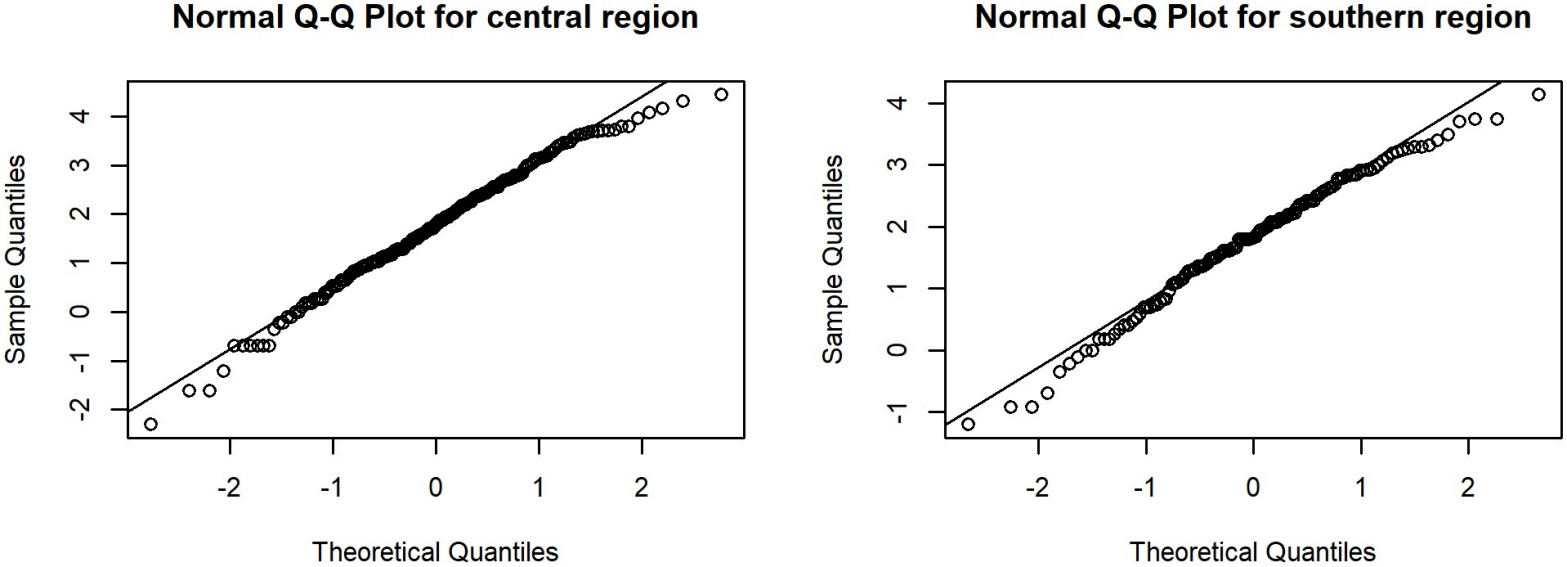
Normal Q-Q plot of log-transformed for rainfall data sets from central and southern regions in Thailand.

As previously mentioned, the CV can be used to measure the dispersion in a dataset, especially in cases like rainfall data that conform to a lognormal distribution with excess zeros. Therefore, daily rainfall data from the central and southern regions (Chumphon province) in August 2017, collected by the Central and Southern Region Irrigation Hydrology Center were used to construct confidence intervals for evaluating the proposed methods. The datasets were shown in Tables 2 and 3. The data with zero values implied a binomial distribution whereas the positive observations for both regions were skewed, as shown in Fig 4, and so log transformation of the positive values was applied. It is imperative to check the distribution of the data, and so a minimum Akaike information criteria (AIC) analysis was conducted. The AIC values of the rainfall data from the central and southern regions for normal, lognormal, and Cauchy distributions were 1491.7130, 1251.7700, and 1390.5430 and 952.6130, 840.1782, and 919.0321, respectively. Thus, the lognormal distribution was suitable for both datasets. This was further confirmed using normal Q-Q plots (Fig 5). The summary statistics for the rainfall data from the central and southern regions were *n*_1_ = 390, ${\hat{\delta }}_{1,1}=0.4667$, ${\hat{\mu }}_{1}=1.7573$, ${\hat{\sigma }}_{1}^{2}=1.6636$, ${\hat{\eta }}_{1}=0.7340$ and *n*_2_ = 248, ${\hat{\delta }}_{2,1}=0.5121$, ${\hat{\mu }}_{2}=1.7913$, ${\hat{\sigma }}_{2}^{2}=1.1872$, ${\hat{\eta }}_{2}=0.6083$, respectively. The ratio of CVs between two regions was *ϕ* = 1.2066, and the 95% confidence intervals for *ϕ* are reported in Table 4.

**Table 2:** Daily rainfall data from central region on August, 2017.

Date	Stations												
	N.67	C.2	C.13	C.30	Ct.4	Ct.5A	Ct.7	Ct.9	Ct.2A	S.9	S.13	S.28	T.7
1	0.0	0.0	0.0	0.0	0.0	0.0	0.0	0.0	0.0	0.0	6.4	0.0	0.0
2	0.0	0.0	0.0	9.2	4.2	0.0	0.0	0.0	17.0	0.0	9.6	2.2	0.0
3	0.0	0.0	0.0	1.1	2.6	0.0	0.0	0.0	0.0	0.0	1.5	0.0	0.0
4	8.2	0.0	0.0	0.0	0.6	0.0	28.5	6.1	1.4	26.2	0.0	0.0	0.0
5	3.9	4.4	36.1	0.0	0.4	0.0	10.0	3.0	0.0	56.3	15.2	8.6	0.0
6	0.0	0.0	2.2	0.0	0.0	10.5	0.0	0.0	1.8	4.1	3.2	4.5	2.9
7	0.0	0.0	0.4	13.5	0.0	19.2	1.0	10.2	4.1	1.5	2.5	1.3	27.9
8	4.0	19.1	0.0	0.0	0.0	7.8	0.0	0.0	3.1	7.8	0.0	0.0	0.0
9	0.0	0.0	0.0	0.0	0.0	0.0	0.0	0.0	0.0	0.0	0.0	0.0	0.0
10	11.0	0.0	17.4	0.0	3.0	0.0	0.0	0.0	0.0	8.0	0.0	0.0	0.0
11	0.0	0.0	0.0	3.8	0.0	0.0	0.0	0.0	0.0	0.0	0.0	0.0	0.0
12	0.0	0.0	9.5	0.0	18.8	0.0	0.0	0.0	0.0	0.0	0.0	0.0	0.0
13	0.0	0.0	0.0	0.0	0.0	0.0	0.0	0.0	0.0	0.0	0.0	0.0	20.0
14	1.3	0.9	0.0	0.0	0.0	0.0	13.5	10.8	0.0	0.0	0.0	0.0	1.1
15	44.2	9.3	10.8	3.4	70.0	0.0	8.3	3.0	52.0	9.3	0.0	0.0	0.0
16	12.0	6.4	26.6	22.2	54.5	54.8	11.5	25.4	9.2	12.3	5.7	2.2	12.4
17	2.6	0.0	9.5	12.5	15.3	65.6	18.5	2.0	9.4	0.0	2.6	12.8	16.2
18	1.0	11.8	12.3	21.6	12.9	14.3	4.0	46.7	0.8	6.2	18.8	6.8	2.2
19	0.0	0.6	44.8	5.1	0.0	0.0	36.5	17.0	14.7	2.1	0.0	26.7	9.5
20	67.8	17.9	3.8	7.6	0.2	17.2	5.5	8.0	9.9	0.0	20.5	0.5	40.0
21	0.0	0.8	0.0	15.4	0.2	6.4	0.0	14.2	0.0	0.0	0.0	0.0	0.0
22	15.0	0.0	0.0	8.5	3.1	0.0	2.2	5.4	0.0	0.0	0.0	0.0	0.0
23	0.0	0.0	0.0	13.9	0.0	8.3	1.5	0.0	0.0	10.0	0.0	0.0	10.5
24	38.5	2.3	24.8	6.5	8.0	5.4	0.0	0.8	30.0	6.2	6.4	0.0	0.0
25	13.0	7.6	0.0	9.3	10.0	10.2	2.0	3.4	0.0	0.0	3.3	0.0	0.0
26	0.0	5.1	0.0	1.9	0.3	7.4	0.0	44.7	0.0	13.6	26.8	26.3	3.9
27	24.7	55.7	12.7	18.2	30.0	0.0	35.0	22.1	13.4	13.0	62.5	4.6	89.9
28	11.0	14.7	0.0	0.0	53.6	10.2	14.0	1.0	7.0	10.6	0.0	2.5	4.9
29	4.8	2.2	0.0	0.0	4.3	15.4	0.0	0.0	21.1	0.0	3.1	0.0	0.0
30	4.4	24.8	0.0	0.0	13.6	0.0	8.0	16.5	19.1	7.3	2.4	5.2	41.9
31	0.0	0.0	0.0	12.7	1.3	0.0	2.0	3.8	0.0	0.0	0.0	0.0	7.2

Source: Central region irrigation hydrology center (http://hydro-5.rid.go.th/)

**Table 3:** Daily rainfall data from southern region on August, 2017.

Date	Stations							
	100191	100251	100261	100271	100281	100291	100301	100311
1	2.0	0.0	4.7	13.6	2.3	2.3	14.7	11.3
2	0.0	0.0	4.8	2.2	3.9	3.9	3.2	0.0
3	6.0	0.0	3.4	4.1	0.0	0.0	8.0	2.0
4	5.0	0.0	2.9	0.0	0.0	0.0	40.8	5.0
5	0.0	0.0	5.3	0.0	0.0	0.0	0.0	0.0
6	0.0	0.0	2.6	0.0	0.0	0.0	13.9	2.1
7	0.0	0.0	0.0	0.0	0.0	0.0	0.0	0.0
8	0.0	0.0	30.0	0.0	0.0	0.0	7.2	1.3
9	0.0	0.0	0.0	0.0	0.0	0.0	0.0	0.0
10	0.0	0.0	0.0	0.0	0.0	0.0	0.0	0.0
11	0.0	0.0	0.0	0.0	0.0	0.0	8.6	0.0
12	0.0	0.0	0.0	0.0	0.0	0.0	0.0	0.0
13	0.0	0.0	0.0	0.0	0.0	0.0	0.0	0.0
14	63.0	10.5	3.6	6.1	42.2	42.2	18.6	10.6
15	0.0	7.0	16.1	22.6	18.3	18.3	1.6	0.0
16	3.0	0.0	17.0	7.4	5.2	5.2	1.8	0.0
17	25.0	5.0	9.0	21.6	17.3	17.3	32.7	27.0
18	16.0	6.0	7.5	16.2	11.3	11.3	20.2	12.8
19	2.0	0.4	7.0	14.0	3.7	3.7	5.0	1.5
20	9.0	0.0	3.1	6.3	8.4	8.4	11.2	1.7
21	0.0	0.0	10.6	3.6	0.0	0.0	0.0	0.0
22	0.0	4.0	6.0	12.2	4.4	4.4	0.3	0.0
23	6.0	0.7	16.9	25.7	0.0	0.0	6.6	1.5
24	0.0	0.8	0.9	24.4	0.0	0.0	0.0	2.1
25	8.0	0.4	19.1	27.1	0.0	0.0	8.0	6.0
26	6.0	0.0	9.2	26.4	0.0	0.0	9.2	3.0
27	1.0	0.5	6.2	13.2	0.0	0.0	3.9	1.2
28	8.0	1.0	27.8	18.7	4.5	4.5	10.0	8.5
29	0.0	1.2	0.0	0.0	0.0	0.0	0.0	1.2
30	0.0	0.0	0.0	0.0	0.0	0.0	1.4	0.0
31	0.0	0.0	12.3	0.0	0.0	0.0	0.0	0.0

Source: Southern region irrigation hydrology center (http://hydro-8.com/main/Submenu/3-RAIN/3-RAIN-02.html)

**Table 4:** The 95% two-sided confidence intervals for the ratio of CVs of daily rainfall data between central and southern region in August, 2017.

Methods	Confidence intervals		
	Lower	Upper	Length
FGCI	1.0540	1.8070	0.7530
B-LIJ (Equitailed)	1.0416	1.8182	0.7766
B-JR (Equitailed)	1.0514	1.7957	0.7443
B-U (Equitailed)	1.0420	1.8296	0.7876
B-LIJ (HPD)	0.9905	1.7545	0.7640
B-JR (HPD)	1.0119	1.7532	0.7412
B-U (HPD)	1.0297	1.8060	0.7762
Wald	1.0316	1.7332	0.7016
Fieller	1.0659	1.7831	0.7172

The lower and upper bounds from the results indicate that the dispersion of rainfall in the central region was more than the southern region. This is because the southern region has abundant precipitation throughout the year due to being located on the peninsula surrounded by the Andaman Sea and the Gulf of Thailand. The central region is located on the plains that cause irregular precipitation, thus the dispersion of the rainfall data is larger than in the southern region.

## Conclusion

FGCI, Bayesian methods based on the left-invariant Jeffreys, Jeffreys rule, and uniform priors, and the Wald and Fieller log-likelihood methods were used to construct the confidence intervals for the ratio of CVs of lognormal distributions with excess zeros. Coverage probabilities and the average lengths were used to evaluate the performance of the proposed methods.

The simulation results indicate that the coverage probabilities for all cases of the FGCI and Bayesian methods using the uniform prior and almost all cases of the Bayesian method using the left-invariant Jeffreys and Jeffreys rule priors were close to or greater than the target. However, when considering the average lengths, the Bayesian method using the Jeffreys rule prior based on the HPD interval produced the shortest ones in cases of small sample sizes and a large sample size together with a small expected number of non-zero observations and a large variance, while FGCI was optimal for the other cases. Therefore, the HPD Bayesian method using the Jeffreys rule prior and the FGCI method are suitable for constructing confidence intervals for the ratio of CVs of lognormal distributions with excess zeros.

Nam and Kwon [16] introduced the Wald-type and Fieller-type methods for the ratio of CVs of lognormal distributions that were appropriate for medium sample sizes. In the present study, this method was extended for a lognormal distribution with zero-inflated observations. However, the coverage probabilities of the Wald log-likelihood method were less than the target for all cases whereas those of the Fieller log-likelihood method were greater than the target for a few cases when the probability of non-zero values was more than half and for a large variance. Moreover, the average lengths of these methods were wider than the FGCI and Bayesian methods. Hence, the Wald and Fieller log-likelihood methods are not recommended for constructing confidence intervals for the ratio of CVs of lognormal distributions with excess zeros.

Furthermore, the confidence intervals evaluation in the empirical study is coincidental with the simulation results.

## References

[1] Thai Meteorological Department. The climate of Thailand. 2015 [cited 1 May 2019]. Available from: https://www.tmd.go.th/en/archive/thailand_climate.pdf.

[2] Fukuchi H. Correlation properties of rainfall rates in the United Kingdom. IEE Proc. Part H (Microwaves, Opt. Antennas). 1988 Apr;135:83–88. doi: 10.1049/ip-h-2.1988.0018

[3] Shimizu KA. bivariate mixed lognormal distribution with an analysis of rainfall data. Amer Meteor Soc. 1993 Feb;32:161–171.

[4] Kong CY, Jamaludin S, Yusof F, Foo HM. Parameter estimation for bivariate mixed lognormal distribution. J Sci Technol. 2012;4:41–48.

[5] Maneerat P, Niwitpong S, Niwitpong S. Confidence intervals for the mean of delta-lognormal distribution. In: Kreinovich V, Sriboonchitta S, Editors. Structural Changes and their Econometric Modeling, Studies in Computational Intelligence. Cham: Springer; 2019a. pp. 264–274.

[6] Maneerat P, Niwitpong S, Niwitpong S. Bayesian confidence intervals for a single mean and the difference between two means of delta-lognormal distributions. Commun Stat-Simul C. 2021;50(10):2906–2934. doi: 10.1080/03610918.2019.1616095

[7] Pennington M. Efficient Estimators of Abundance, for Fish and Plankton Surveys. Biometrics. 1983 Mar;39(1):281–286. doi: 10.2307/2530830

[8] Lo NC, Jacobson LD, Squire JL. Indices of relative abundance from fish spotter data based on delta-lognormal models. Can J Fish Aquat Sci. 1992 Jun;49:2515–2526. doi: 10.1139/f92-278

[9] Ingram GW Jr, Richards WJ, Lamkin JT, Muhling B. Annual indices of Atlantic bluefin tuna (*Thunnus thynnus*) larvae in the Gulf of Mexico developed using delta-lognormal and multivariate models. Aquat Living Resour. 2010;23:35–47. doi: 10.1051/alr/2009053

[10] Owen WJ, DeRouen TA. Estimation of the mean for lognormal data containing zeroes and left- censored values, with applications to the measurement of worker exposure to air contaminants. Biometrics. 1980 Dec;36:707–719. doi: 10.2307/2556125

[11] Tian L, Wu J. Confidence intervals for the mean of lognormal data with excess zeros. Biom J. 2006;48:149–156. doi: 10.1002/bimj.200510155 16544820

[12] Callahan CM, Kesterson JG, Tierney WM. Association of symptoms of depression with diagnostic test charges among older adults. Ann Intern Med. 1997;126:426–432. doi: 10.7326/0003-4819-126-6-199703150-00002 9072927

[13] Chen Y-H, Zhou X-H. Generalized confidence intervals for the ratio or difference of two means for lognormal populations with zeros. UW Biostatistics Working Paper Series. 2006 Sep; Working Paper 296.

[14] Verrill S, Johnson RA. Confidence bounds and hypothesis tests for normal distribution coefficients of variation. Commun Stat-Theory Methods. 2007 Aug;36(12):2187–2206. doi: 10.1080/03610920701215126

[15] Gulhar M, Kibria BMG, Albatineh AN, Ahmed NU. A comparison of some confidence intervals for estimating the population coefficient of variation: a simulation study. SORT-Stat Oper Res T. 2012 Jan;36:45–68.

[16] Nam J, Kwon D. Inference on the ratio of two coefficients of variation of two lognormal distributions. Commun Stat-Theory Methods. 2017 May;46(17):8575–8587. doi: 10.1080/03610926.2016.1185118

[17] Marek M. Practical application of coefficient of variation. XIII Congreso Internacional en Energía y Recursos Minerales. 2013. Available online: https://www.researchgate.net/publication/275648121_Practical_application_of_coefficient_of_variation (accessed on 18 November 2019).

[18] Wong ACM, Wu J. Small sample asymptotic inference for the coefficient of variation: normal and nonnormal models. J Stat Plan Inference. 2002;104:73–82. doi: 10.1016/S0378-3758(01)00241-5

[19] Mahmoudvand R, Hassani H. Two new confidence intervals for the coefficient of variation in a normal distribution. J Appl Stat. 2009 Apr;36(4):429–442. doi: 10.1080/02664760802474249

[20] Hayter AJ. Confidence bounds on the coefficient of variation of a normal distribution with applications to win-probabilities. J Stat Comput Simul. 2015;85(18):3778–3791. doi: 10.1080/00949655.2015.1035654

[21] Hasan MS, Krishnamoorthy K. Improved confidence intervals for the ratio of coefficients of variation of two lognormal distributions. J Stat Theory Appl. 2017 Sep;16(3):345–353. doi: 10.2991/jsta.2017.16.3.6

[22] Wong A, Jiang L. Improved small sample inference on the satio of two coefficients of variation of two independent lognormal distributions. J Probab Stat. 2019 Mar. Available online: 10.1155/2019/7173416 (accessed on 20 September 2019).

[23] Sangnawakij P, Niwitpong S. Confidence intervals for functions of coefficients of variation with bounded parameter spaces in two gamma distributions. Songklanakarin J Sci Technol. 2017a Jan;39:27–39.

[24] Sangnawakij P, Niwitpong S. Confidence intervals for coefficients of variation in two-parameter exponential distributions. Commun Stat-Simul Comput. 2017b Apr;46(8):6618–6630. doi: 10.1080/03610918.2016.1208236

[25] Buntao N, Niwitpong S. Confidence intervals for the difference of coefficients of variation for lognormal distributions and delta-lognormal distributions. Appl Math Sci. 2012;6(134):6691–6704.

[26] Buntao N, Niwitpong S. Confidence intervals for the ratio of coefficients of variation of delta-lognormal distribution. Appl Math Sci. 2013;7(77):3811–3818.

[27] Yosboonruang N, Niwitpong S, Niwitpong S. Confidence intervals for the coefficient of variation of the delta-lognormal distribution. In: Anh L, Le DS, Kreinovich V, Thach NN, Editors. Econometrics for Financial Applications, Studies in Computational Intelligence. Cham: Springer; 2018. pp. 327–337.

[28] Yosboonruang N, Niwitpong S, Niwitpong S. Confidence intervals for coefficient of variation of three parameters delta-lognormal distribution. In: Kreinovich V, Sriboonchitta S, Editors. Structural Changes and their Econometric Modeling, Studies in Computational Intelligence. Cham: Springer; 2019a. pp. 352–363.

[29] Yosboonruang N, Niwitpong S, Niwitpong S. Measuring the dispersion of rainfall using Bayesian confidence intervals for coefficient of variation of delta-lognormal distribution: a study from Thailand. PeerJ. 2019b Jul; 7:e7344. doi: 10.7717/peerj.7344 31367487 PMC6657683

[30] Aitchison J. On the distribution of a positive random variable having a discrete probability and mass at the origin. J Am Stat Assoc. 1955 Sep; 50(271):901–908. doi: 10.1080/01621459.1955.10501976

[31] Fisher RA. Inverse probability. Math Proc Camb Philos Soc. 1930 Oct;26(4):528–535. doi: 10.1017/S0305004100016297

[32] Hannig J. On generalized fiducial inference. Stat Sin. 2009;19:491–544.

[33] Li X, Zhou X, Tian L. Interval estimation for the mean of lognormal data with excess zeros. Stat Probab Lett. 2013;83:2447–2453. doi: 10.1016/j.spl.2013.07.004

[34] Dawid AP, Stone M. The functional-model basis of fiducial inference. Ann Stat. 1982;10:1054–1067. doi: 10.1214/aos/1176345970

[35] Aldrich J. Fisher’s “inverse probability” of 1930. Int Stat Rev. 2000;68(2):155–172. doi: 10.2307/1403666

[36] Hannig J, E L, Abdel-Karim A, Iyer H. Simultaneous fiducial generalized confidence intervals for ratios of means of lognormal distributions. Aust J Stat. 2006a;35(2&3):261–269.

[37] Hannig J, Iyer H, Patterson P. Fiducial generalized confidence intervals. J Am Stat Assoc. 2006b Mar;101(473):254–269. doi: 10.1198/016214505000000736

[38] Jeffreys H. An invariant form for the prior probability in estimation problems. Proc R Soc Lond A. 1946;186:453–461. doi: 10.1098/rspa.1946.0056 20998741

[39] Ghosh JK, Delampady M, Samanta T. An introduction to Bayesian analysis: theory and methods. New York: Springer;2006.

[40] Harvey J, van der Merwe AJ. Bayesian confidence intervals for means and variances of lognormal and bivariate lognormal distributions. J Stat Plan Infer. 2012;142:1294–1309. doi: 10.1016/j.jspi.2011.12.006

[41] Stone JV. Bayes’ Rule: a tutorial introduction to Bayesian analysis. Sheffield: Sebtel Press;2013.

[42] O’Reilly JX, Mars RB. Bayesian models in cognitive neuroscience: a tutorial. In: Forstmann BU, Wagenmakers E-J, Editors. An introduction to model-based cognitive neuroscience. New York: Springer; 2015. pp. 179–197.

[43] Bolstad WM, Curran JM. Introduction to Bayesian statistics. New Jersey: John Wiley & Sons;2017.

[44] Kalkur TA, Rao A. Bayes estimator for coefficient of variation and inverse coefficient of variation for the normal distribution. International Journal of Statistics and Systems. 2017;12(4):721–732.

[45] Fieller EC. Some problems in interval estimation. J R Stat Soc Ser B Methodol. 1954;16(2):175–185.

[46] Wu WH, Hsieh HN. Generalized confidence interval estimation for the mean of delta-lognormal distribution: an application to New Zealand trawl survey data. J Appl Stat. 2014;41(7):1471–1485. doi: 10.1080/02664763.2014.881780

